# Structural basis for urate recognition and apigenin inhibition of human GLUT9

**DOI:** 10.1038/s41467-024-49420-9

**Published:** 2024-06-12

**Authors:** Zilin Shen, Li Xu, Tong Wu, Huan Wang, Qifan Wang, Xiaofei Ge, Fang Kong, Gaoxingyu Huang, Xiaojing Pan

**Affiliations:** 1 Beijing Frontier Research Center for Biological Structure, Tsinghua-Peking Joint Center for Life Sciences, State Key Laboratory of Membrane Biology, School of Life Sciences, Tsinghua University, 100084 Beijing, China; 2 Institute of Bio-Architecture and Bio-Interactions (IBABI), Shenzhen Medical Academy of Research and Translation (SMART), Shenzhen, 518107 Guangdong China; 3 Westlake Laboratory of Life Sciences and Biomedicine, Key Laboratory of Structural Biology of Zhejiang Province, School of Life Sciences, Westlake University, Hangzhou, 310024 Zhejiang China; 4 Institute of Biology, Westlake Institute for Advanced Study, Hangzhou, 310024 Zhejiang China

**Keywords:** Cryoelectron microscopy, Membrane proteins, Structural biology

## Abstract

Urate, the physiological form of uric acid and a potent antioxidant in serum, plays a pivotal role in scavenging reactive oxygen species. Yet excessive accumulation of urate, known as hyperuricemia, is the primary risk factor for the development of gout. The high-capacity urate transporter GLUT9 represents a promising target for gout treatment. Here, we present cryo-electron microscopy structures of human GLUT9 in complex with urate or its inhibitor apigenin at overall resolutions of 3.5 Å and 3.3 Å, respectively. In both structures, GLUT9 exhibits an inward open conformation, wherein the substrate binding pocket faces the intracellular side. These structures unveil the molecular basis for GLUT9’s substrate preference of urate over glucose, and show that apigenin acts as a competitive inhibitor by occupying the substrate binding site. Our findings provide critical information for the development of specific inhibitors targeting GLUT9 as potential therapeutics for gout and hyperuricemia.

## Abstract (web summary)

Uric acid is a serum antioxidant, but excess can cause gout. Authors investigated GLUT9, crucial for urate reabsorption, and determined its structure with urate and apigenin providing insight for substrate preference and rational drug design.

## Introduction

Gout, characterized by the deposition of monosodium urate crystals^1,2^, is the most prevalent form of inflammatory arthritis, and affects a substantial population of approximately 41 million adults worldwide^3,4^. Hyperuricemia is the leading cause of gout and often associated with other metabolic diseases, such as chronic kidney diseases, diabetes, and adverse cardiovascular effects^5–8^. The development of hyperuricemia can be attributed to dysregulation in purine metabolism, urate excretion, and reabsorption. Intervention of any of these steps may afford the opportunity for the treatment of hyperuricemia^9,10^.

GLUT9, encoded by *SLC2A9*, was initially identified as a glucose transporter for its sequence similarity with GLUT1-4^11^ (Supplementary Fig. 1). Subsequent genome-wide association scans highlighted the crucial role of GLUT9 as a high-capacity urate uniporter^12–16^. GLUT9 demonstrates an evident preference for urate over glucose, with the transport activity for urate 45 to 60 times higher than for glucose^15^.

The N terminus of GLUT9 plays a pivotal role in protein trafficking. Consequently, the full-length (FL) GLUT9 is predominantly located on the basolateral membrane and functions as the sole transporter responsible for the efflux of urate from the cell into the blood. In contrast, the amino terminus-deleted GLUT9 (GLUT9ΔN) is targeted to the apical membrane in renal tubules, and serves as one of the major transporters for the reabsorption of urate from the renal tubular lumen into the cell^16–20^. Dysfunction of GLUT9 results in almost complete blockage of urate reabsorption^15,21–24^ (Supplementary Table 1).

In Supplementary Table 2, various inhibitors capable of binding with GLUT9 are listed. Notably, among these inhibitors, apigenin (API) emerges as a standout candidate with a less binding targets and a higher binding affinity in comparison to others. API, a natural flavonoid compound with high abundance in herbs such as celery and chamomile, acts as a competitive inhibitor for GLUT9 mediated urate transport^25,26^. When administered in the context of hyperuricemia nephropathy, API effectively ameliorates kidney damage by reducing urate levels and alleviating renal inflammation and fibrosis^26^. Nevertheless, the development of an effective and safe drug targeting GLUT9 for gout treatment remains a challenge due to the lack of selective and potent inhibitors. High-resolution structures of GLUT9 in complex with substrates or lead compounds may provide critical insights for the rational drug design or optimization.

GLUT9 is a member of the major facilitator superfamily (MFS)^11,27^. The core fold of all MFS transporters possesses twelve transmembrane helices (TMs) that are arranged into two distinct domains, the amino-terminal domain (NTD) and the carboxy-terminal domain (CTD). Each domain encompasses six TMs that fold into a pair of “3 + 3” inverted repeats, with the three-helix bundle serving as the structural and functional unit^28–32^ (Supplementary Fig. 1). Structures of GLUT1, GLUT3, GLUT4, and GLUT5 have revealed the molecular details for monosaccharide binding and the alternating access transport mechanism^33–36^. Although the fold of GLUT9 can be predicted with confidence, the molecular basis for specific recognition of urate and API requires high-resolution structures.

In this study, we present the cryo-electron microscopy (cryo-EM) structures of human GLUT9 bound with urate and API, which unravel the molecular determinants for urate preference over glucose and reveal the inhibitory mechanism of API on GLUT9. These findings not only advance our understanding of the substrate binding and transport mechanism of GLUT9, but also offer important clue to the development of GLUT9-targeting drugs.

## Results

### Functional characterization of recombinantly expressed human GLUT9

The coding sequence of *SLC2A9* was amplified from the cDNA library of HEK293F and inserted into a pCAG expression vector, containing tandem FLAG and strep affinity tags at the N terminus. Prior to cryo-EM analysis, we conducted a functional characterization to test the transport activity of the recombinantly expressed GLUT9.

**Fig. 1:**
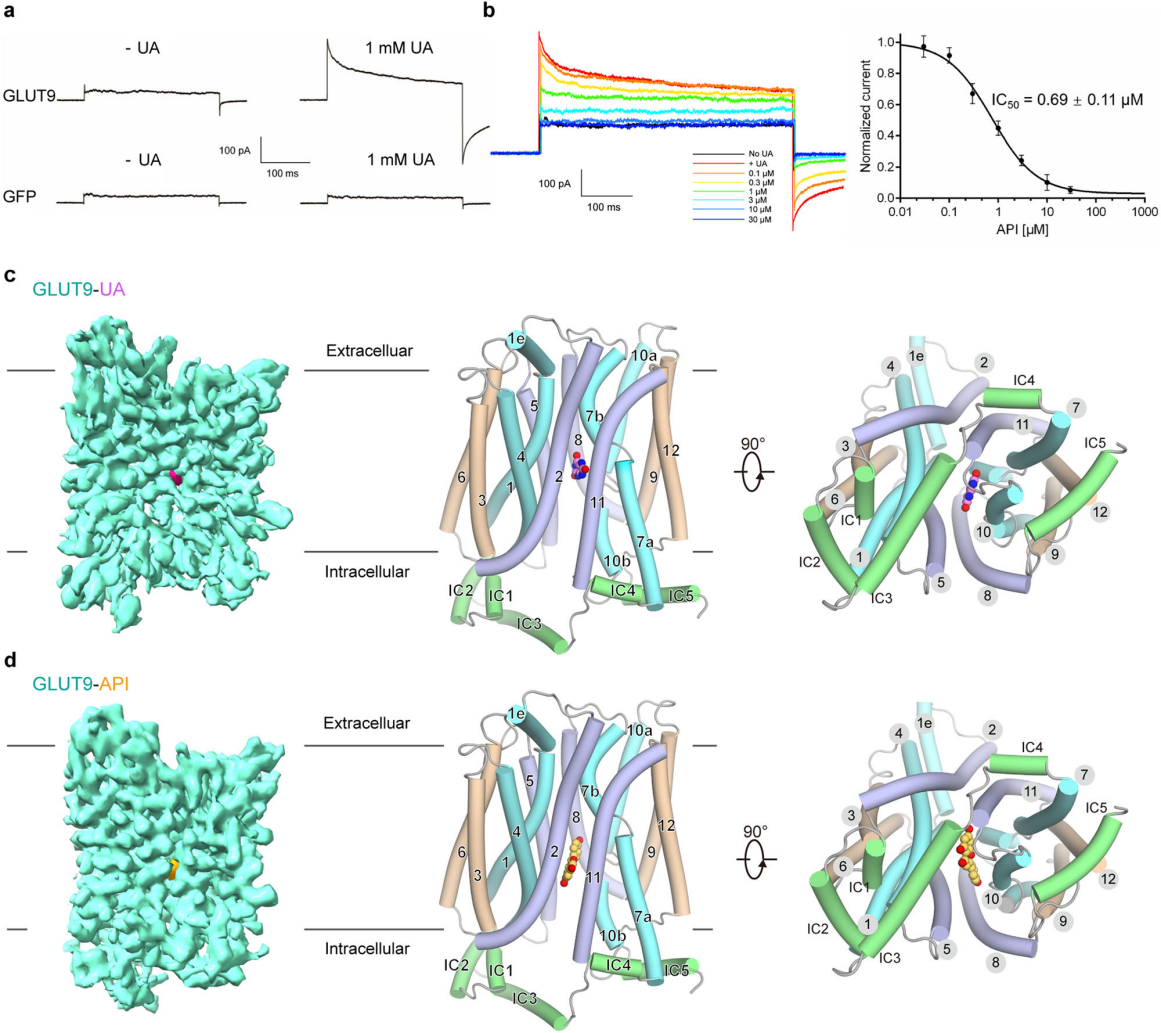
Cryo-EM analysis of human GLUT9 in complex with the substrate urate and a natural product inhibitor API. **a** Electrophysiological characterization of human GLUT9 transiently expressed in HEK293T cells. Representative currents, elicited by the transport of urate (UA), are recorded for human GLUT9 (top) or the control protein GFP (bottom). The holding potential is −30 mV, and the current was elicited at 30 mV for 300 ms before repolarized to −30 mV. **b** Inhibition of urate transport activity of GLUT9 by API. Left: Representative currents of GLUT9 in the presence of 1 mM UA or API applied at indicated concentrations. Right: IC_50_ of API is shown. Data represent mean ± SEM. *n* is the cell number recorded, and *n* = 5, 4, 5, 8, 8, 8 and 6 for API concentrations at 0.03, 0.1, 0.3, 1, 3, 10 and 30 μM, respectively. Concentration response curve was fitted with *Y* = Bottom + (Top − Bottom) / (1 + 10^((LogIC_50_ −*X*) * Hill Slope)). **c**, **d** Overall structures of GLUT9-UA and GLUT9-API. Identical views of the two complexes, both are in the inward-open conformation, are shown in three presentations. Left: Cryo-EM maps of the complexes, with GLUT9 protein, UA and API colored cyan, pink and yellow, respectively. Middle and Right: a side view and cytoplasmic view of the overall structures. UA and API are shown as pink and yellow spheres, respectively. For visual clarity, the corresponding TM segments in the four 3-helix repeats are colored the same. The intracellular (IC) helices are colored green. All structure figures, if not otherwise indicated, were prepared in PyMol^61^.

Transport of urate, which carries a negative charge, across the cell membrane could be monitored using patch clamp techniques^37,38^ (Fig. 1a). Indeed, in the presence of 1 mM urate, current was recorded for HEK293T cells overexpressing GLUT9, but not GFP, when voltage was applied. These currents can be blocked by commercially obtained API with an IC_50_ value of 0.69 ± 0.11 μM (Fig. 1b).

### Cryo-EM analysis of GLUT9

For structural determination using cryo-EM, GLUT9 was recombinantly overexpressed in HEK293F cells following the protocol described in the Methods. However, the protein eluted in the buffer at pH 7.4 from affinity purification was prone to aggregation during concentration. Inspired by our previous trials for the purification of voltage-gated sodium channels human Na_v_1.4 and NaChBac, we conducted a pH titration ranging from 6.0 to 8.0 with 0.5 increments^39–41^. To our satisfaction, GLUT9 no longer aggregated during concentration at pH 6.0 (Supplementary Fig. 2a).

When we set out to prepare cryo-samples with the purified protein, there came another technical obstacle. The protein particles exhibited preferred orientation in the vitreous ice on carbon coated gold grids. To solve this issue, we screened several types of grids combined with different detergents. Ultimately, proteins purified in 0.005% (w/v) lauryl maltose neopentyl glycol (LMNG) plus 0.0005% cholesteryl hemisuccinate Tris salt (CHS) dispersed in multiple orientations on gold grids coated with amorphous nitinol support film (Supplementary Fig. 2b, c).

Based on the abovementioned functional characterizations, we incubated purified GLUT9 with 5 mM urate or 1 mM API for a duration of 30 min prior to cryo-sample preparation. Following a routine protocol similar to that for the cryo-EM data acquisition and image processing of human GLUT4 (Supplementary Fig. 3), we successfully obtained three dimensional (3D) EM reconstructions of GLUT9 bound with urate or API with overall resolutions of 3.5 Å and 3.3 Å, respectively (Fig. 1c, d and Supplementary Figs. 3–5, Supplementary Table 3).

The excellent cryo-EM map immediately suggested a similar inward-open conformation of both complexes. Model building was thus facilitated by docking the AlphaFold predicted human GLUT9 with manual adjustment. Briefly, GLUT9 observes the canonical MFS fold for the twelve TMs. The NTD and the CTD display a two-fold pseudo symmetry, enclosing a central cavity that opens to the intracellular side, confirming the inward-open state^28,33,35^(Fig. 1c, d and Supplementary Fig. 6). After structural assignment of the polypeptide chain, additional densities were found in the central cavity in both EM maps. We therefore built urate and API to these densities and hereafter name these two structures GLUT9-UA and GLUT9-API for description simplicity (Fig. 1c, d and Supplementary Fig. 5b). Both the binding poses between urate or API and GLUT9 from the cryo-EM structures were initially verified using binding pose metadynamics (BPMD) simulations. The PoseScore provided by BPMD for GLUT9-UA and GLUT9-API, which are 1.78 and 2.16 Å, respectively, indicates that the ligands have been accurately modeled and are well-refined in the cryo-EM structures (Supplementary Fig. 7a, b).

### Molecular basis for the specific recognition of urate by GLUT9

**Fig. 2:**
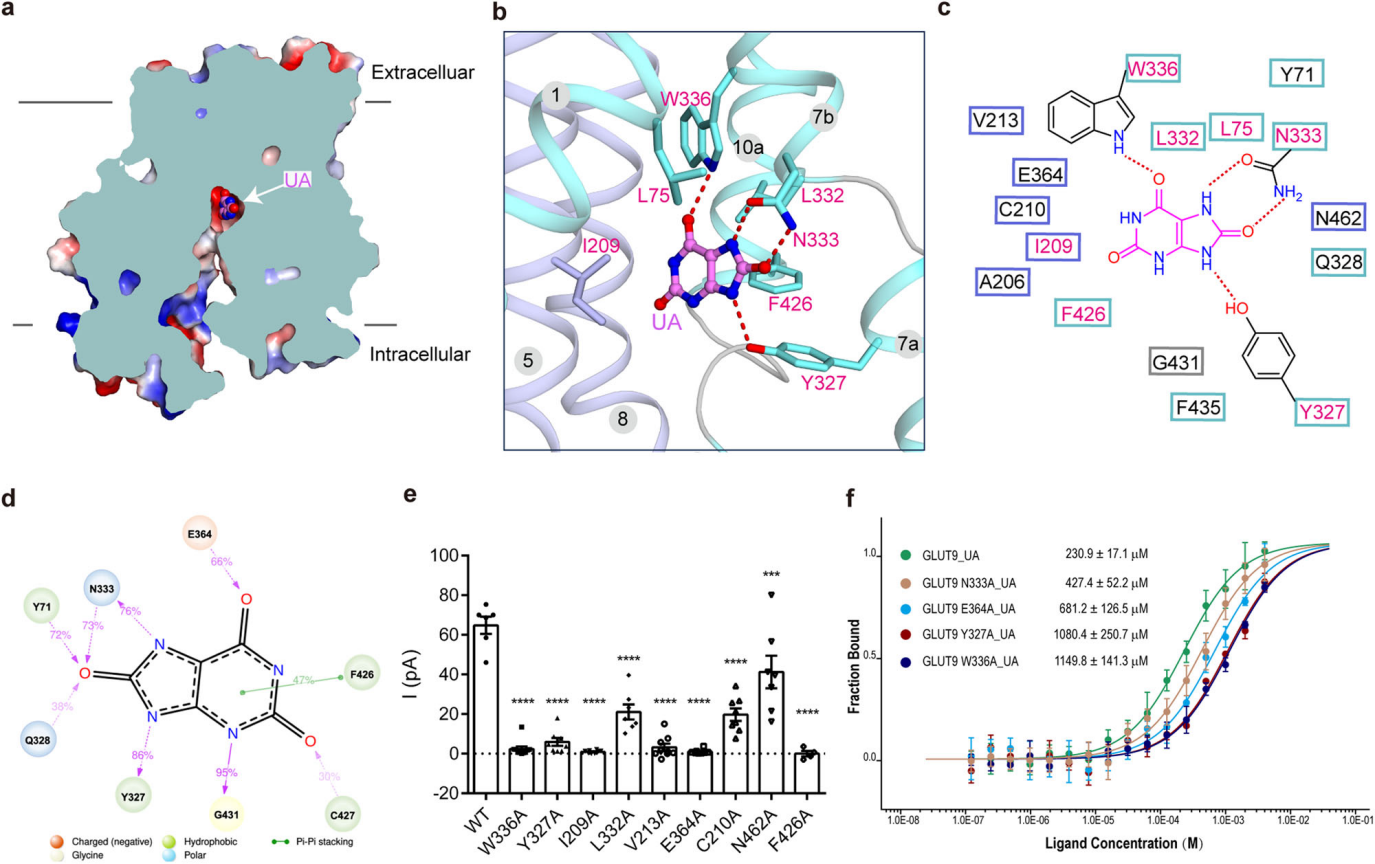
Structural basis for urate recognition by GLUT9. **a** The cut-open representation of the surface electrostatic potential of GLUT9-UA complex. **b** Coordination of UA, shown as pink ball and sticks, by central cavity residues. The residues in direct contact with UA are shown as sticks. **c** Plane diagram of residues constituting the binding pocket within the cut-off distance from UA of 5 Å. The direct H-bonds are indicated by red dashed lines in (**b**, **c**). And this is applied in all the figures for interaction analysis. **d** H-bond and π–π interactions between GLUT9 and urate were maintained for more than 30% throughout the simulation. **e** Whole-cell patch clamp recordings were performed to validate the residues involved in urate binding. *n* is the number of experimental cells from which recordings were obtained. And *n* = 6, 10, 8, 8, 7, 9, 8, 8, 7, 4 for wildtype (WT), W336A, Y327A, I209A, L332A, V213A, E364A, C210A, N462A, F426A, respectively. Data are mean ± SEM. ***p = 0.0001 versus WT, ****p < 0.0001 versus WT. Statistical significance was assessed using one-way ANOVA analysis. **f** Microscale thermophoresis (MST) measurement about the binding affinities of GLUT9 variations with urate. *n* represents the replicates number of the experiment, and *n* = 6, 6, 5, 3, 4 for WT, N333A, E364A, Y327A, W336A, respectively. Data represent mean ± SEM.

Urate binds to the central cavity of GLUT9 (Fig. 2a). The complex structure reveals extensive interactions between GLUT9 and the substrate urate. Notably, W336, N333 and Y327 form hydrogen bonds (H-bonds) with urate, while L75, I209, and L332 establish hydrophobic interactions (Fig. 2b, c). Additionally, there are some van der Waals contacts from V213, E364, C210, A206 and N462 (Fig. 2c). The molecular dynamics (MD) simulation based analysis also reveals the interaction frequency, which reinforces the presence of H-bonds and hydrophobic interactions (Fig. 2d). Specifically, during the simulation, N333 acts as both a proton donor (73% of the simulation time) and a proton acceptor (76% of the simulation time) for urate. Y327 forms a hydrogen bond with urate during 86% of the simulation time.

To further validate the significance of these binding residues, substitutions were made by replacing them with alanine. Subsequently, electrophysiology experiments in HEK293T cells and microscale thermophoresis (MST) experiments with purified proteins were conducted to evaluate the effects. Almost all the substitutions resulted in well-expressed proteins with significantly reduced ion currents in electrophysiology and decreased binding affinity in MST (Fig. 2e, f and Supplementary Fig. 8). In the MST results, N333A, E364A, Y327A, and W336A exhibited much lower binding affinity, with values ranging from 230.9 ± 17.1 µM to 472.4 ± 52.2 µM, 681.2 ± 126.5 µM, 1080.4 ± 250.7 µM and 1149.8 ± 141.3 µM, respectively. Notably, mutations in N333 and L75 were found to have specific implications for hypouricemia, resulting in a loss of transport activity of GLUT9^22,37,42^. L75 variations affected protein expression and trafficking, which were validated in our imaging experiments (Supplementary Fig. 8). These findings from electrophysiological experiments, MST results combined with the clinical manifestations of hypouricemia (Supplementary Table 1), provide supports to the structural coordination between GLUT9 and urate.

### Structural basis for substrate preference of urate over glucose in GLUT9

**Fig. 3:**
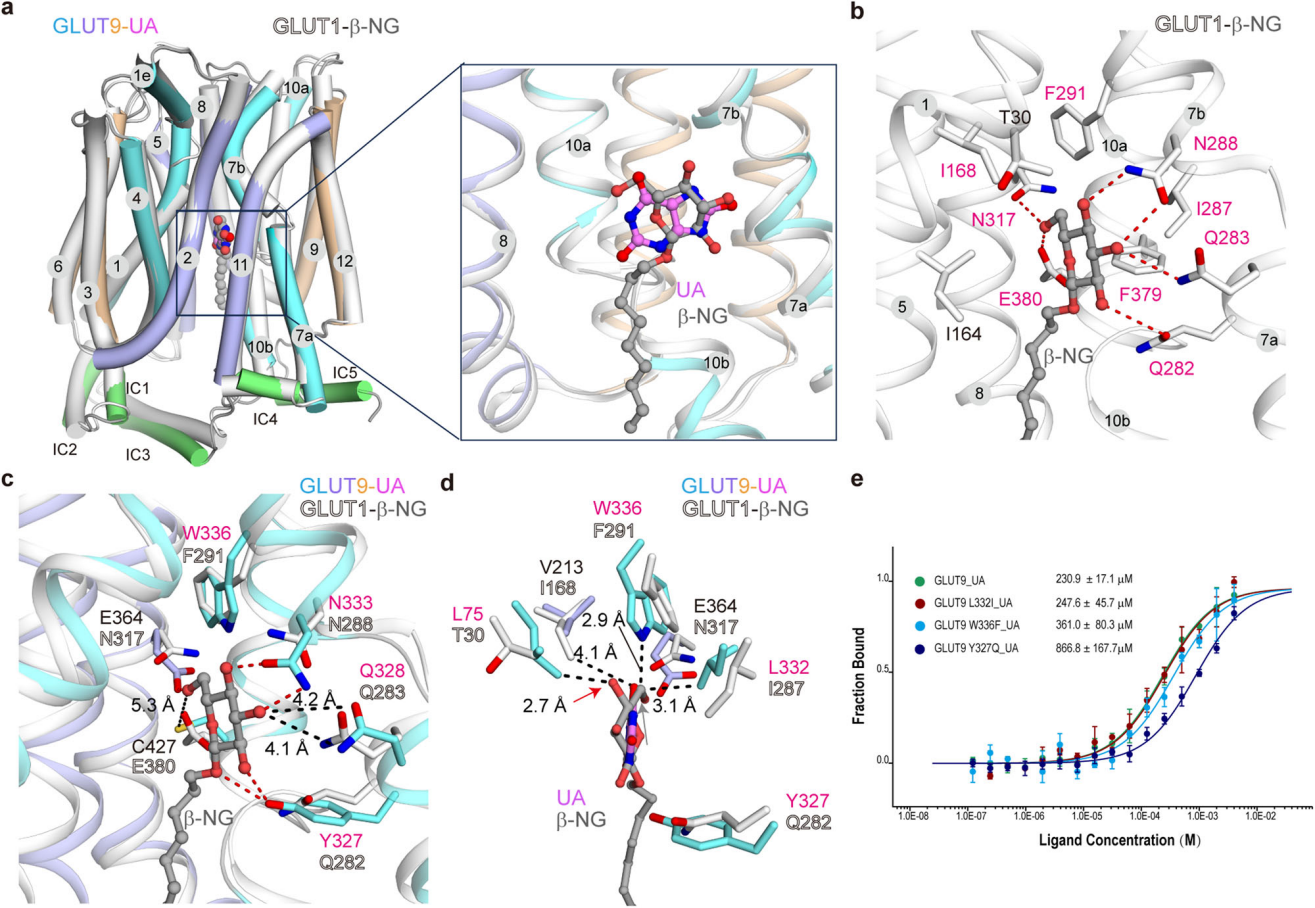
Molecular basis for GLUT9’s substrate preference of urate over glucose. **a** Urate and glucose bind to similar pockets of GLUT9 and GLUT1, respectively. Shown here are the superimposed structures of GLUT9-UA and GLUT1-n-nonyl-β-D-glucopyranoside (β-NG) (PDB code: 4PYP) align with the C domain. The root mean square deviation (RMSD) is 1.24 Å among 396 Cα atoms. GLUT1 and β-NG are shown in white and gray, respectively. **b** Details of β-NG recognition by GLUT1 in the same view as Fig. 2b. **c** The different binding residues of GLUT9 and GLUT1 underlie the substrate preference of urate over glucose. The potential H-bonds between GLUT9 and β-NG are indicated by red dashed lines. The distances between the amino group and the acyl group on the sidechain of GLUT9 Q328 and β-NG are 4.1 Å and 4.2 Å, respectively. The corresponding residue of E380 in GLUT1 is replaced by C427 in GLUT9, the sidechain of which is about 5.3 Å from β-NG. **d** The central pocket of GLUT9 exhibits limited compatibility for β-NG recognition. For visual clarity, the conserved residues between GLUT9 and GLUT1 are hidden. The distance between GLUT9 L75 Cδ and the β-NG atom, indicated by a red arrow, is 2.7 Å. Similarly, the distances between the GLUT9 W336 amide group and GLUT9 L332 Cδ with the β-NG atom, marked by gray arrows, are 2.9 Å and 3.1 Å, respectively. These distances suggest the presence of slight clashes between GLUT9 and glucose. The distance between GLUT1 I168 Cδ and β-NG are 4.1 Å, substitution with V213 in GLUT9 would result in the loss of the hydrophobic interaction. The red and gray arrows are corresponding to one hydroxyl group and the methylene group of β-NG, respectively. **e** MST experiment measures the binding affinity of urate with substitutions of GLUT9 with counterparts from GLUT1. *n* represents the replicates number of the experiment, and *n* = 6, 3, 3, 5 for WT, L332I, W336F, Y327Q, respectively. Data represent mean ± SEM.

While GLUT1 exclusively transports glucose, GLUT9 exhibits the ability to transport glucose, fructose, adenine and urate, with much higher capacity for the latter^14,15,43^. By conducting structure-guided sequence analysis, we made interesting discoveries regarding this preference. Superimposition of the structures of GLUT9-UA and GLUT1-n-nonyl-β-D-glucopyranoside (β-NG) (PDB code: 4PYP) reveals nearly identical structures with the similar binding pockets, except for minor changes of the intracellular helices (IC1-5) (Fig. 3a and Supplementary Fig. 6).

In the GLUT1-β-NG complex, the coordination of glucose involves several H-bonds with Q282, Q283, N288, N317 and E380, as well as hydrophobic interactions with I168, F291, I287, and F379 (Fig. 3b). A comparison of the binding pockets of GLUT1-β-NG and GLUT9-UA highlights distinct residues in GLUT9 that may lose potential interactions with glucose (Fig. 3c, d). Specifically, the replacement of E380 in GLUT1 with C427 in GLUT9 leads to the loss of H-bond formation with D-glucose (Fig. 3c). Similarly, GLUT1-I168 establishes hydrophobic interactions with β-NG, while its corresponding residue V213 in GLUT9, which has a shorter sidechain, almost loses this interaction (Fig. 3d).

The distances between the amino group and the acyl group on the sidechain of GLUT9 Q328 and β-NG are 4.1 Å and 4.2 Å, respectively, indicating that this residue on GLUT9 or the substrate glucose may undergo conformational changes to form H-bonds with D-glucose (Fig. 3d and Supplementary Fig. 9). In GLUT9, the distance between L75 Cδ and β-NG is 2.7 Å. Similarly, the distances between W336 amide group, L332 Cδ and the methylene group of β-NG are 2.9 Å and 3.1 Å, respectively. These observations suggest that glucose binding in the central pocket of GLUT9 could potentially result in slight clashes, unless the sidechains of the residues or glucose itself undergo conformational changes (Supplementary Fig. 9). However, other positions on GLUT9, including Y327, N333, and E364, which correspond to Q282, N288, and N317 in GLUT1, can still form interactions with D-glucose (Fig. 3c). Therefore, a partial retention of stable coordination likely accounts for GLUT9’s ability to transport glucose, albeit at a significantly lower capacity (45–60 times) compared to urate^15^.

To further validate this preference mechanism, substitutions of GLUT9 were made with counterpart residues of GLUT1. MST experiments with purified proteins were conducted to evaluate the effects. L332I has little effect on urate binding, while W336F and Y327Q exhibited much lower binding affinity, with values ranging from 230.9 ± 17.1 µM to 361.0 ± 80.3 µM and 866.8 ± 167.7 µM, respectively (Fig. 3e). The notably reduced binding affinities suggest the critical role of W336 and Y327 in determining the preference.

Conversely, examining the residues implicated in urate recognition in GLUT9 and comparing them to GLUT1 reveals a dearth of H-bonds between GLUT1 and urate (Supplementary Fig. 10). This lack of H-bonds is likely the primary factor preventing GLUT1 from recognizing urate.

Additionally, the stability of the GLUT9 complex differs when bound to urate compared to glucose, as evidenced by the root mean square fluctuation (RMSF) analysis (Supplementary Fig. 11a, b). The predicted binding residues exhibit lower RMSF values, indicating that their stability is maintained through the protein-ligand interaction. These interactions involve H-bonds, hydrophobic interactions, and water-mediated H-bonds, as depicted in Supplementary Fig. 11c, d. Notably, the direct hydrogen bonds are more extensive in the GLUT9-UA complex compared to the GLUT9-GLU (docking model of GLUT9 and glucose) complex (Supplementary Fig. 11c, d). The number of contacts formed between GLUT9 with urate is much more than that between GLUT9 and glucose during the simulation time (Supplementary Fig. 11e, f). These combined findings offer a possible rationale for GLUT9’s substrate preference for urate over glucose.

### GLUT9 blockade by API

**Fig. 4:**
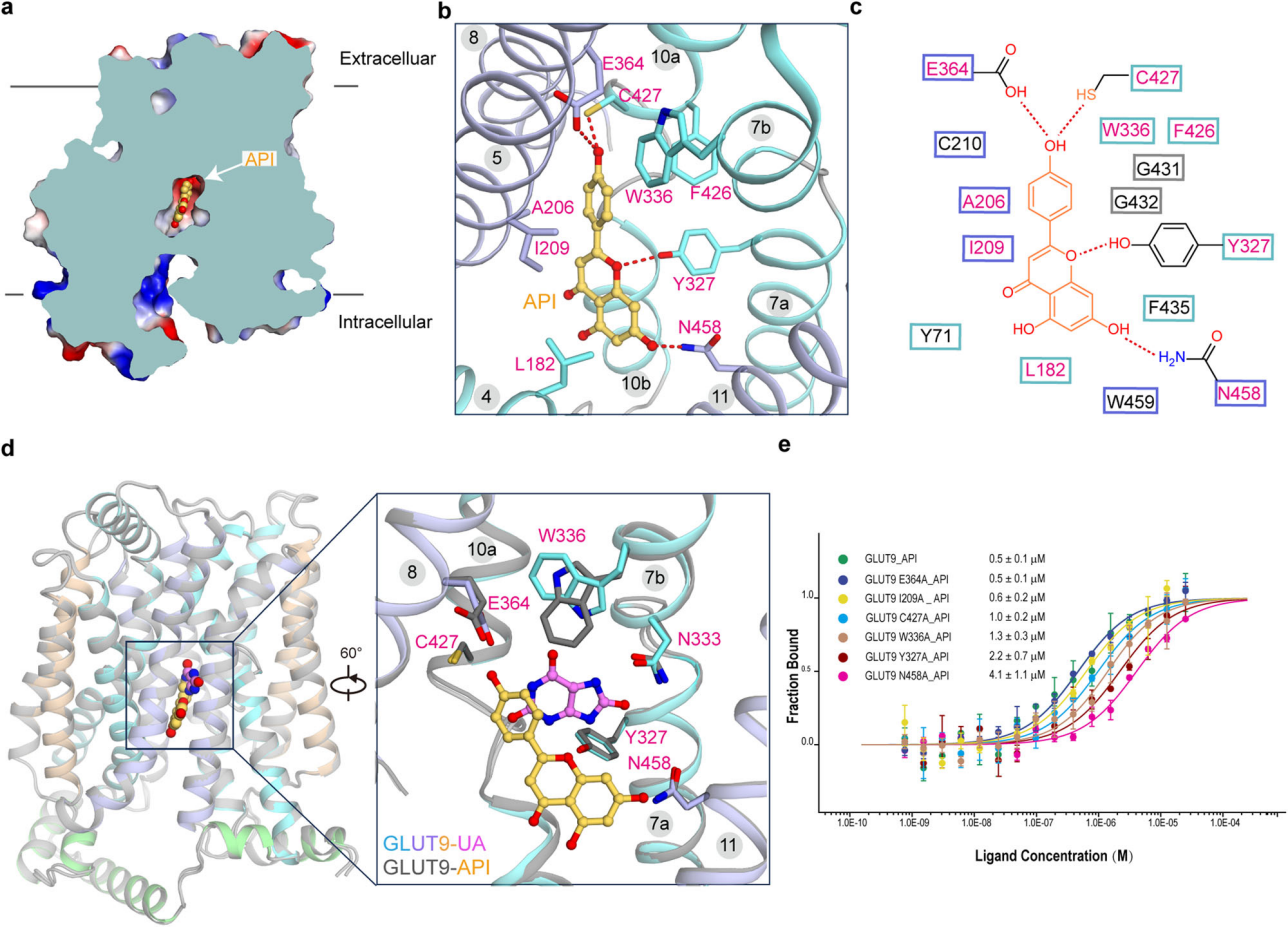
API occupies the substrate binding pocket in the inward-open conformation of GLUT9. **a** The cut-open representation of the surface electrostatic potential. **b** Coordination of API, shown as yellow ball and sticks, by central cavity residues. The residues in direct contact with API are shown as sticks. **c** Plane diagram of residues constituting the binding pocket within the cut-off distance from API of 5 Å. **d** The only change in the central cavity between urate and API bound GLUT9 structures is the flip of the side chain of W336. API bound GLUT9 is shown in gray. The RMSD is 0.35 Å among 423 Cα atoms. **e** MST measurement of the affinities of GLUT9 variations with API. *n* represents the replicates number of the experiment, and *n* = 3, 3, 3, 3, 8, 3, 3 for WT, E364A, I209A, C427A, W336A, Y327A, N458A, respectively. Data represent mean ± SEM.

The cryo-EM density corresponding to the bound API was clearly resolved within the central cavity of GLUT9 (Fig. 4a and Supplementary Fig. 5b). The orientation of entire molecule aligned along the transport path (Fig. 4a). The API was stabilized by polar interactions, including H-bonds formed by E364, C427, Y327, and N458, as well as hydrophobic interactions with F426, I209, A206, and L182, which surround the carbon backbone of the inhibitor. Additionally, W336 formed a π-π interaction with the API in the structure (Fig. 4b, c). To access whether the binding of the inhibitor induced any conformational changes, the structure was compared with that of GLUT9-UA (Fig. 4d). The superimposition revealed nearly identical backbone structures, with the only disparity observed in W336, whose side chain had to flip to avoid steric clashes while establishing a H-bond with the substrate urate (Fig. 4d). To identify the residues that play an essential role in API binding and gain insights for drug optimization, MST experiments were conducted. N458A, Y327A, W336A, and C427A significantly decreased the binding affinity, with values ranging from 0.5 ± 0.1 µM to 4.1 ± 1.1 µM, 2.2 ± 0.7 µM, 1.3 ± 0.3 µM and 1.0 ± 0.2 µM, respectively (Fig. 4e). However, I209A and E364A had little effect on API binding. Notably, while both C427 and E364 were predicted to have H-bonds with the API in the structure, MST examination showed that E364A had almost no effect on the binding. This suggests that C427 plays a more critical role in H-bond formation.

## Discussion

**Fig. 5:**
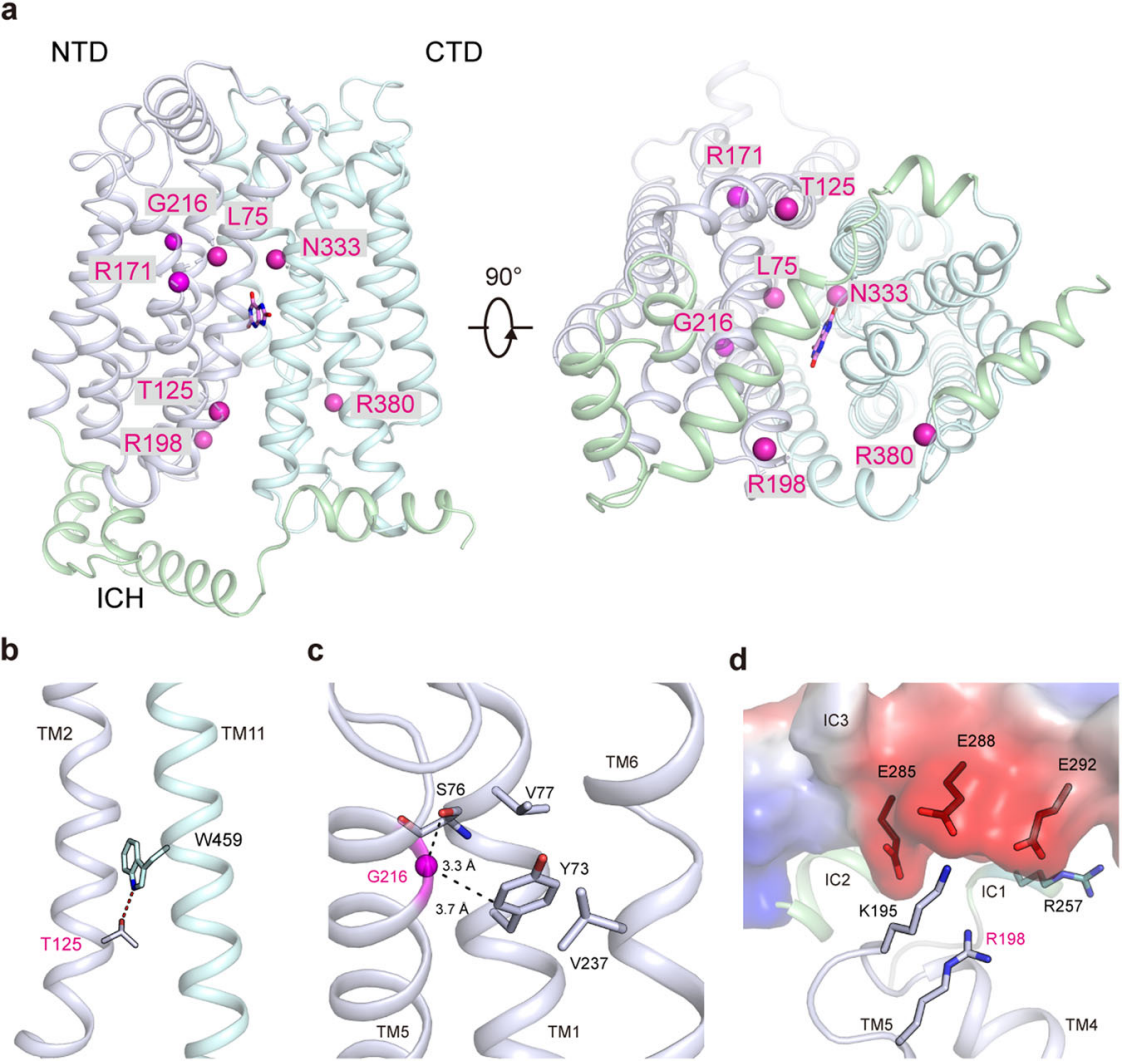
Structural interpretation of representative GLUT9 mutations related to hypouricemia. **a** Disease mutations derived from Type 2 renal hypouricemia (RHUC2) are colored magenta on the structure of GLUT9-UA complex. The NTD, CTD, ICH and urate are colored blue-white, pale cyan, pale green and pink, respectively. **b** H-bond is formed between T125 and W459, which may be involved in NTD and CTD interactions during alternating access cycle. **c** Detailed coordination of G216 on TM5 and the residues in proximity to the G216 Cα atom. The Cα atom of G216 is represented as magenta sphere. The distances between the G216 Cα atom and the ketonic oxygen of S76 mainchain, and the Cδ atom of Y73 are 3.3 Å and 3.7 Å, respectively. **d** Electrostatic interactions between R198 and acidic residues on the IC3 helix.

The structures of human GLUT9 provide reliable templates for the analysis of disease-related mutations (Fig. 5a and Supplementary Table 1)^21,22,37,44,45^, offering valuable insights into the mechanisms underlying specific diseases and potential strategies for intervention. Loss of function mutations on GLUT9 abolish kidney urate reabsorption, leading to Type 2 renal hypouricemia (RHUC2)(Supplementary Table 1). The disease-derived mutations in transporter proteins can be broadly categorized into several groups, including those responsible for substrate recognition, state transitions during transportation which is known as alternating access cycle^28,34,46^, and protein folding and trafficking.

In GLUT9, as previously mentioned, N333 play crucial roles in substrate recognition^22,37^. The T125M mutation, for instance, could potentially disrupt the formation of H-bonds with W459, consequently impacting the interaction between the NTD and CTD, ultimately influencing protein folding (Fig. 5b). It is consistent with functional validation about the protein expression^37^. The distance between G216 Cα and the ketonic oxygen on mainchain of S76, as well as Y73 Cδ, is measured at 3.3 Å and 3.7 Å, respectively (Fig. 5c). This suggests that replacing G216 with a much bulkier arginine residue would introduce steric clashes within the N-domain fold, thereby influencing protein expression and trafficking^37^. R198, located on the intracellular side of TM5, engages in electronic interactions with acidic residues on the IC3 helix (Fig. 5d). The ICH domain acts as a latch that tightens the intracellular gate in the outward conformation^33,35^. The disruption of this interaction primarily affects the gating during the alternating access cycle, leading to a reduction in transport activity. This mutation, derived from RHUC2, is consistent with both disease-associated mutations in GLUT1 deficiency syndrome and results observed in GLUT4 transportation assays (Fig. 5d)^33,35,37^. Likewise, R380 on TM9 potentially have interaction with IC5, as the mutation R380W leads to reduced activity^37^. Furthermore, the conservation of these two arginine residues across the GLUT transporters strongly suggests their indispensable role in the alternating access cycle (Supplementary Fig. 1).

In summary, this study sheds light on the molecular mechanisms underlying the substrate preference for urate over glucose and the inhibitory effects of API on GLUT9. These findings not only deepen our understanding of these processes but also offer opportunities for drug optimization, specifically in improving the selectivity and potency of API inhibition. The implications of this research extend to potential therapeutic approaches for addressing conditions like gout and hyperuricemia, paving the way for further investigations in this field.

## Methods

### Transient expression of human GLUT9

The full length human *SLC2A9* gene (Gene ID: 56606) was cloned from HEK293F cDNA, and was recombined into pCAG vector with twin strep and one FLAG tag at the amino terminus^47^. The HEK293F cells (Invitrogen) cultured with SMM 293-TII medium (Sino Biological), under the condition of 37 °C, 5% CO_2_ in a Multitron-Pro shaker (Infors, 130 r.p.m.) were transfected with the expression plasmids when the cell density reached 1.5 ~ 2.0 × 10^6^ cells per ml. About 2.0 mg plasmids were pre-incubated with 4 mg 40-kDa linear polyethylenimines (PEIs, Yeasen) in 20 ml fresh medium for 15 min before adding into 1 L of cell culture. Transfected cells were cultured in the same condition for another 48 h before harvesting.

### Protein purification

6 L cells transfected with GLUT9 plasmids were harvested by centrifugation at 800 *g* and resuspended in the lysis buffer containing 25 mM MES, 150 mM NaCl, pH 6.0. The suspension was extracted at 4 °C with 1% (w/v) n-dodecyl-β-D-maltopyranoside (DDM, Anatrace), 0.1% (w/v)  CHS, and protease inhibitors containing 2 mM phenylmethylsulfonyl fluoride (PMSF, Amresco), aprotinin (6.5 μg/mL, MedChemExpress), pepstatin (3.5 μg/mL, Sigma), and leupeptin (25 μg/mL, Sigma). After 3 h extraction, the cell lysate was applied to centrifugation and the supernatant was double loaded onto anti-Flag M2 affinity gel (Sigma) column. The resin was washed using the W buffer (25 mM MES at pH 6.0, 150 mM NaCl, 3.5 μg/mL pepstain, 6.5 μg/mL aprotinin, 25 μg/mL leupeptin, 0.005% (w/v) LMNG plus 0.0005% CHS) and the protein was eluted with W buffer plus 0.4 mg/mL FLAG peptide. The elution was then loaded onto Strep-Tactin Sepharose (IBA), washed using W buffer and eluted with W buffer plus 2.5 mM D-Desthiobiotin (IBA). Finally, the elution was concentrated to about 1 mL with a 30 kDa cut-off Centricon (Millipore) and then loaded on superose-6 column (GE Healthcare) for size-exclusion chromatography in the W buffer. The fractions with our protein detected by both the UV absorption and SDS-PAGE staining (Supplementary Fig. 2a) were collected and concentrated to about 1 mg/mL. 1 mM API (final concentration) was added to the purified protein and incubated for 0.5 h, then the mixture was used for cryo-EM sample preparation. For GLUT9-UA complex, 1 mM urate exists in all the buffers throughout the entire process of protein purification, and extra 5 mM of urate was added before preparing the cryo-EM sample.

### Whole-cell patch-clamp recording of GLUT9 in HEK293T cells

The patch clamp protocol for recording GLUT9 current refers to the characterization study in 2021 by Chen et al.^38^. HEK293T cells were transfected with plasmids of human GLUT9 with eGFP at the C terminus using lipo2000 (Invitrogen). After 24–48 h, cells with green fluorescence were imaged with confocal setup and clamped to record urate current.

The bath solution contained 140 mM NaCl, 5 mM KCl, 1 mM MgCl_2_, 2 mM CaCl_2_, 10 mM HEPES, and 10 mM glucose (pH adjust to 5.5 with NaOH), notably, this special pH is suggested by two transport activity papers which showed pH 5.5 was much better than pH 7.4^38,43^. The borosilicate pipettes (Sutter Instrument) had a resistance of 2-4 MΩ and were filled with the internal solution composed of 140 mM KCl, 1 mM MgCl_2_, 5 mM EGTA, and 10 mM HEPES (pH adjusted to 7.4 with KOH). Currents were elicited with a voltage step from −30 mV to +30 mV, using an EPC10-USB amplifier with Patchmaster software v2*90.2 (HEKA Electronic), filtered at 3 kHz (low-pass Bessel filter) and sampled at 50 kHz. In the absence of urate, base current (I_base_) was recorded. Then the cell was incubated with 1 mM urate till the urate-induced current (I_induce_) was stable. Current through GLUT9 (I_GLUT9_) was calculated as: I_GLUT9_ = I_induce_–I_base_.

To investigate the effect of API on urate current, cells were firstly perfused with bath solution and followed with bath solution containing 1 mM urate, to measure the I_GLUT9_.Then bath solution containing 1 mM urate and different concentrations of API was perfused to the recording cells till the current was stably blocked (I_block_). Inhibition effect of API were calculated as: (I_block_ − I_base_) / I_GLUT9_. Concentration-response curve was fitted with: *Y* = Bottom + (Top − Bottom) / (1 + 10^((LogIC_50_ − *X*) * Hill Slope)), where IC_50_ is the concentration of API that blocks 50% of the current. *X* is log of API concentration, and Hill Slope is the slope factor. Data was analyzed using Origin (OriginLab) and GraphPad Prism (GraphPad Software). All data points are presented as mean ± SEM and *n* is the number of experimental cells from which recordings were obtained.

### Cryo-EM data acquisition

Gold grids coated with amorphous nitinol support film (M01 GD-NiTiA31213, Nanodim Technology Limited) were used, with 35 s glow-discharge under the medium power of Plasma Cleaner PDC-32G (Harrick). Aliquots of 3.5 μL freshly purified GLUT9 with urate or API were placed on the grids and were blotted for 4.5 s before plunge frozen in liquid ethane cooled by liquid nitrogen with Vitrobot Mark IV (Thermo Fisher) under the condition of 8 °C and 100% humidity. Electron micrographs were acquired on a Titan Krios electron microscope (Thermo Fisher) operating at 300 kV and equipped with a Gatan K3 Summit detector and GIF Quantum energy filter. A total of 8961 movie stacks for urate bound (GLUT9-UA) and 11,900 movie stacks for API bound (GLUT9-API) were automatically collected using AutoEMation^48^ with a slit width of 20 eV on the energy filter and a preset defocus ranging from −1.8 µm to −1.5 µm in super-resolution mode. Each stack was exposed for 2.56 s with 0.08 s per frame, resulting in 32 frames per stack. The total dose rate was 50 e^−^/Å^2^ for each stack. The stacks were motion-corrected with MotionCor2^49^ and binned 2-fold, resulting in 1.0979 Å/pixel and 1.0825 Å/pixel for GLUT9-UA and GLUT9-API, respectively. Dose weighting was performed for both data^50^. The defocus values were estimated using Gctf^51^.

### Image processing

All the data processing was carried out in cryoSPARC^52^. A diagram for data processing of GLUT-API is presented in Supplementary Fig. 3. For the GLUT9-API complex, 6359 micrographs were collected at begining, after first round of template picking and 2D classification, 1,494,149 particles were selected to do 3D reconstruction, and an initio map of 4.18 Å were resolved. This map was then used for the second round of template picking. In total, 1,282,010 particles were selected for the heterogeneous refinement and 3D classification, a good set of 150,905 particles was selected and refined a map of 3.81 Å. At this stage, we could see some sidechains of GLUT9, but the resolution was not high enough. Then, we collected another 5541 micrographs and did the template picking and 2D classification as mentioned above. Through merging all good particles and removing duplicates, 2,033,696 particles were singled out. After one round of heterogeneous refinement, 357,943 particles were selected, which could refine a map of 3.54 Å. Using 2D classification to remove particles that are not particularly good, we finally refined a map of 3.28 Å. A similar approach was used for data processing of GLUT9-UA. To sum up, 8961 micrographs were collected with the pixel size of 1.0979 Å. After two rounds of template picking, 2D classification, heterogeneous refinement and 3D classification, a 3.51 Å map was refined (Supplementary Figs. 3–5 and Supplementary Table 3).

### Model building and structure refinement

Model building was performed based on the maps of GLUT9 in the presence of urate or API. The starting model of GLUT9, which was predicted by AlphaFold2, was fitted into the EM maps by Chimera^53^. All GLUT9 residues were manually checked in COOT^54^. The chemical properties of amino acids were taken into consideration during model building. In total, 467 residues were assigned with sidechains (Supplementary Table 3). The unmodelled segments include the N-terminal 53 residues and the C-terminal sequences after A520.

Structural refinement was performed using phenix.real_space_refine application in PHENIX^55^ real space with secondary structure and geometry restraints. Over-fitting of the overall model was monitored by refining the model in one of the two independent maps from the gold-standard refinement approach and testing the refined model against the other map^56^. Statistics of the map reconstruction and model refinement can be found in Supplementary Table 3.

### Molecular docking simulation

Protein preparation: the structures of GLUT9-UA and GLUT1 (PDB code: 4PYP)^33^ were refined and prepared in Schrödinger Maestro v13.5 ^57^. A restrained minimization using the Impref module of Impact with the optimized potential liquid simulation (OPLS4) force field^58^ was employed to minimize hydrogen atoms while allowing for sufficient heavy atom movement to relax strained bonds, angles, and clashes^59^.

Ligand preparation: the two ligands (glucose and urate) were prepared using Ligprep to generate the Low-energy, all-atom 3D conformers^60^. The potential ionization states for each ligand structure were generated at a physiological pH of 6.5 ± 1 and minimized using an OPLS4 force field.

Docking simulation: the ligand glucose was docked against GLUT9. All dockings were performed using the Glide Dock in Maestro with the “Extra precision (XP) mode” with more accuracy^61^. The docked conformers were assessed using the Glide (G) Score.

### Molecular dynamics (MD) simulations

Unlike docking, which provides a static viewpoint on how a molecule binds to a protein’s active site, MD simulation calculates the movement of atoms over time using Newton’s classical equation of motion. In this study, the metadynamics simulations were employed to assess the binding poses of urate and API to GLUT9 in the Cryo-EM structures by analyzing the stability of the ligand binding to protein. Classic molecular dynamics (MD) simulations, on the other hand, were used to examine the interactions between urate or glucose with GLUT9.

System setup: the protein was incorporated into a palmitoyl-oleoyl phosphatidylcholine (POPC) bilayer. The resultant system was solvated using the transferable intermolecular interaction potential three points (TIP3P) water model with an orthorhombic box. The system was then neutralized by adding counterions of K^+^ and Cl^−^ ions at a concentration of 150 mM.

Metadynamics simulations: the metadynamics method implemented in Maestro was utilized for the binding poses^62^. Each system underwent ten individual metadynamics simulations, each lasting 10 ns. The collective variable (CV) used to determine the Root Mean Square Deviation (RMSD) of the ligand’s heavy atoms from their initial position was employed in this measure. To calculate RMSD, alignment was performed using protein residues within a 3 Å proximity of the ligand. The hill height and width were set at 0.05 kcal/mol (about one-tenth of the system’s typical thermal energy, kBT) and 0.02 Å, respectively. Before the metadynamics run, the system was immersed in a container of TIP3P water molecules and underwent a series of minimization and restrained MD steps. These steps helped achieve a gradual attainment of the desired temperature of 300 K and alleviate any unfavorable contacts or structural strain present in the initial configuration. Finally, the snapshot from the brief, unbiased MD simulation lasting 0.5 ns was used as the starting point for the subsequent metadynamics production phase.

Classic MD simulations: after the systems were set up, the models were put into a relaxed state prior to the simulation. The simulations were run in Maestro for 100 ns with the NpT ensemble at a temperature of 300 K and a pressure of one atmosphere.

### Microscale thermophoresis (MST)

The binding affinities of GLUT9 variants with urate were assessed by incubating purified protein at a concentration of 300 nM with urate at 16 different concentrations ranging from 4 mM to 0.000122 mM in the W buffer (25 mM MES at pH 6.0, 150 mM NaCl, 3.5 μg/mL pepstain, 6.5 μg/mL aprotinin, 25 μg/mL leupeptin, 0.005% (w/v) LMNG plus 0.0005% CHS). Samples were loaded into MO-Z0025 capillaries (Nano-Temper Technologies) at room temperature for 10 min. MST analyses were conducted using a Monolith NT. Label-free instrument with software MO.Control v1.6 (Nano-Temper Technologies GmbH) at 25 °C with LED power set at 20% and MST power at 40%. Similarly, the binding affinities of GLUT9 variants with API were determined. Purified protein at 30 nM were mixed with ligand API concentrations ranging from 25 µM to 0.000763 µM with serial dilutions in the W buffer supplemented with 0.1% Pluronic F127 (Sigma-Aldrich, St. Louis, MO) and 0.1% DMSO. After a 10 min incubation at room temperature, samples were loaded into MO-Z0025 capillaries (Nano-Temper Technologies) for analysis. MST measurements were conducted at 25 °C using LED power set at 80% and MST power at 40%. Each assay was repeated three to eight times, and *K*_d_ values were calculated using the MO. Affinity Analysis v.2.2.4 software.

### Reporting summary

Further information on research design is available in the Nature Portfolio Reporting Summary linked to this article.

### Supplementary information

Supplementary Information

Peer Review File

Reporting Summary

### Source data

Source Data

## Data Availability

The data that support this study are available from the corresponding authors upon request. The cryo-EM maps have been deposited in the Electron Microscopy Data Bank (EMDB) under accession codes EMD-38966 (human GLUT9-UA) and EMD-38968 (human GLUT9-API). The atomic coordinates have been deposited in the Protein Data Bank (PDB) under accession codes 8Y65 (human GLUT9-UA) and 8Y66 (human GLUT9-API). The source data underlying Figs. 1a, 2e, f, 3e and 4e are provided as a Source Data file. Initial/Final structures from MD simulations are included in the Source Data zip folder as.txt files. Source data are provided with this paper.
