# Supplementary material for: Structural basis for urate recognition and apigenin inhibition of human GLUT9
Source: Nat Commun. 2024 Jun 12;15:5039. doi: 10.1038/s41467-024-49420-9 (PMC11169512; doi:10.1038/s41467-024-49420-9)
Supplement: Supplementary file 1 — Supplementary Information [file 41467_2024_49420_MOESM1_ESM.pdf]

Supplementary information

Structural basis for urate recognition and apigenin inhibition of human GLUT9

Zilin Shen[1,5], Li Xu[2,5], Tong Wu[1,5], Huan Wang[1,5], Qifan Wang[1], Xiaofei Ge[1], Fang Kong[1], Gaoxingyu Huang[3,4], and Xiaojing Pan[2,6]

[1]Beijing Frontier Research Center for Biological Structure, Tsinghua-Peking Joint Center for Life Sciences, State Key Laboratory of Membrane Biology, School of Life Sciences, Tsinghua University, Beijing 100084, China

[2]Institute of Bio-Architecture and Bio-Interactions, Shenzhen Medical Academy of Research and Translation, Shenzhen 518107, Guangdong Province, China

[3]Westlake Laboratory of Life Sciences and Biomedicine, Key Laboratory of Structural Biology of Zhejiang Province, School of Life Sciences, Westlake University, Hangzhou 310024, Zhejiang Province, China

[4]Institute of Biology, Westlake Institute for Advanced Study, Hangzhou 310024, Zhejiang Province, China

[5]These authors contributed equally to this work.

[6]To whom correspondence should be addressed: X. Pan (panxj@smart.org.cn).

This PDF file includes:

Supplementary Figs. 1-11

Supplementary Tables 1-3

Supplementary Figures and Legends

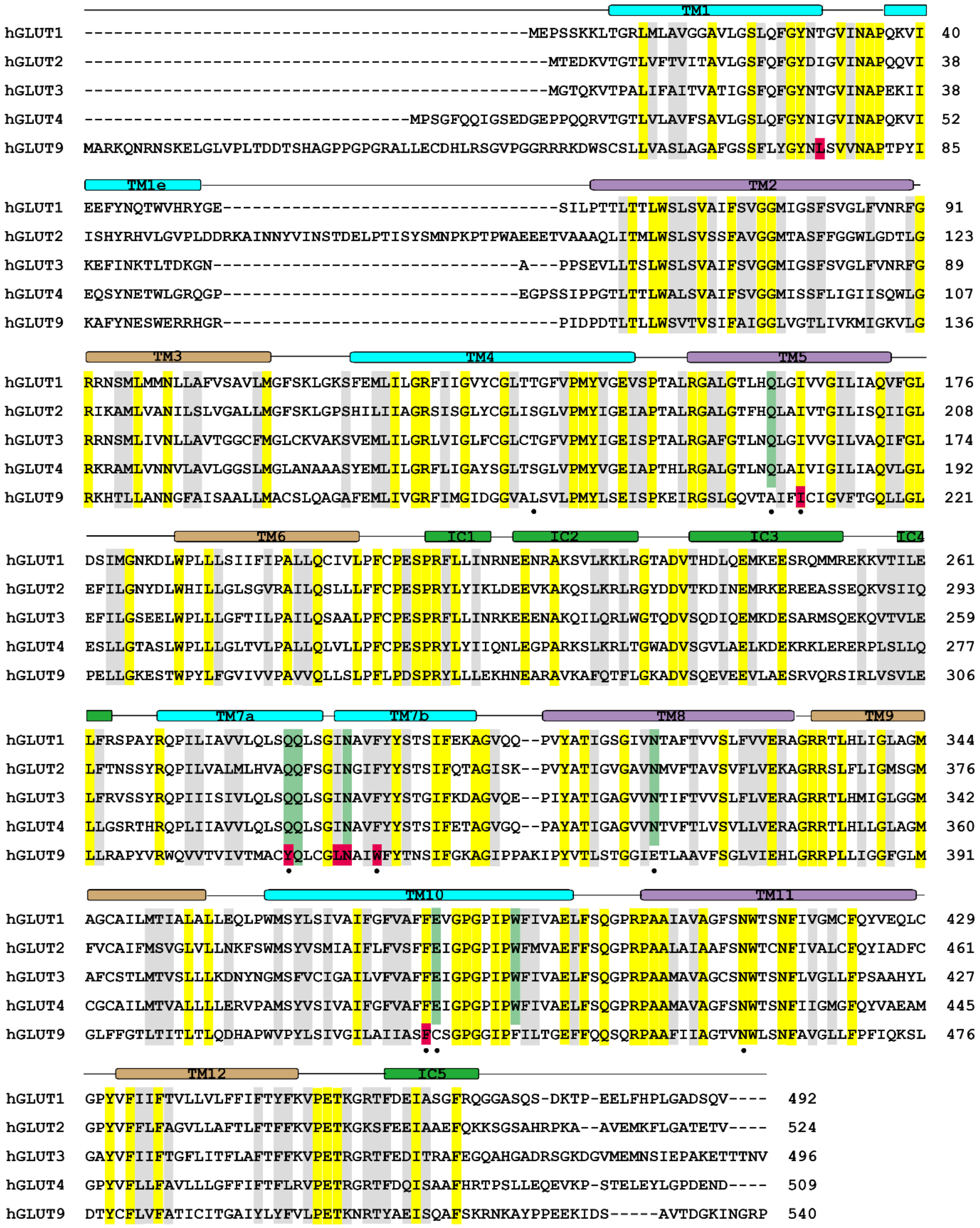

Supplementary Fig. 1 | Sequence alignment of human GLUT9 with glucose transporters GLUT1-4. The sequences were aligned using Clustal Omega [1]. Secondary structural elements of human GLUT9 are indicated above the sequences and color-coded for TMs as in the structure. Invariant residues are shaded yellow, and the conserved ones are shaded gray. The residues involved in urate (UA) and glucose binding are shaded red and green, respectively. The residues that were examined for apigenin (API) blockage are highlighted with black dots under the sequences. The Uniprot IDs for the aligned sequences are: GLUT1: P11166; GLUT2: P11168; GLUT3: P11169; GLUT4: P14672; GLUT9: Q9NRM0.

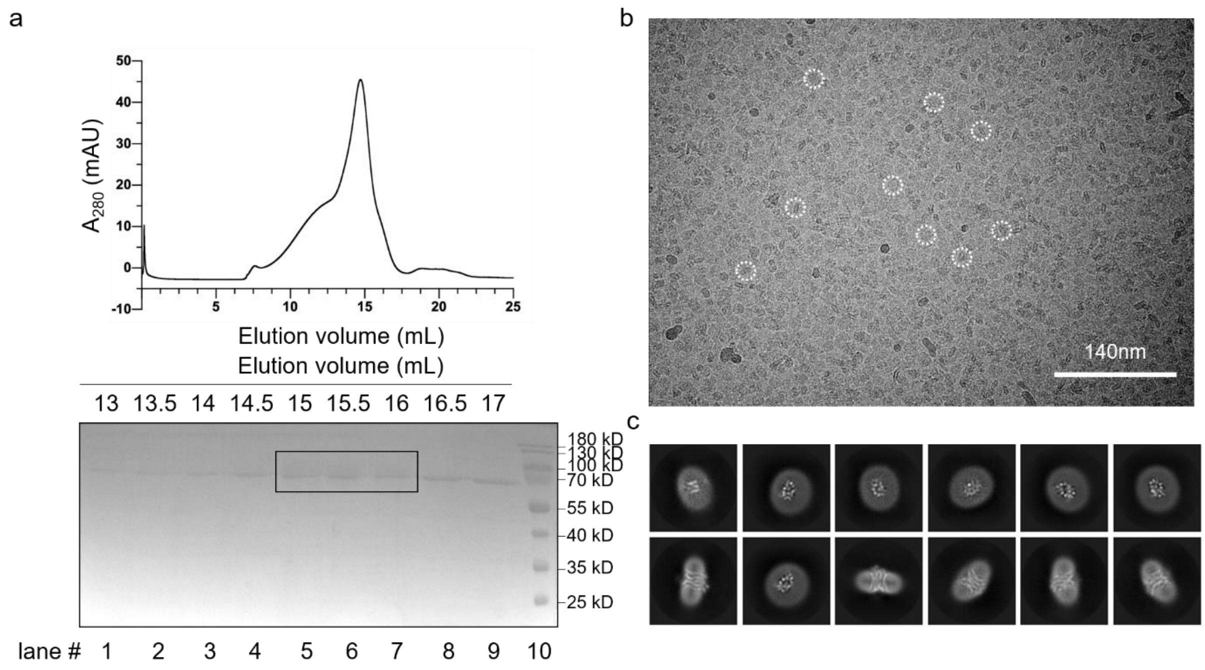

Supplementary Fig. 2 | Structural determination of human GLUT9. **a,** Last step purification of GLUT9. Shown here is a representative size-exclusion chromatogram (SEC) for GLUT9 proteins recombinantly expressed in HEK293F cells. Coomassie-blue-stained SDS-PAGE for corresponding SEC fractions is shown below. The fractions indicated by black box were concentrated for cryo sample preparation. **b,** A representative cryo-EM micrograph. Representative particles in distinct orientations are highlighted with white dotted circles. **c,** Representative 2D classifications of human GLUT9.

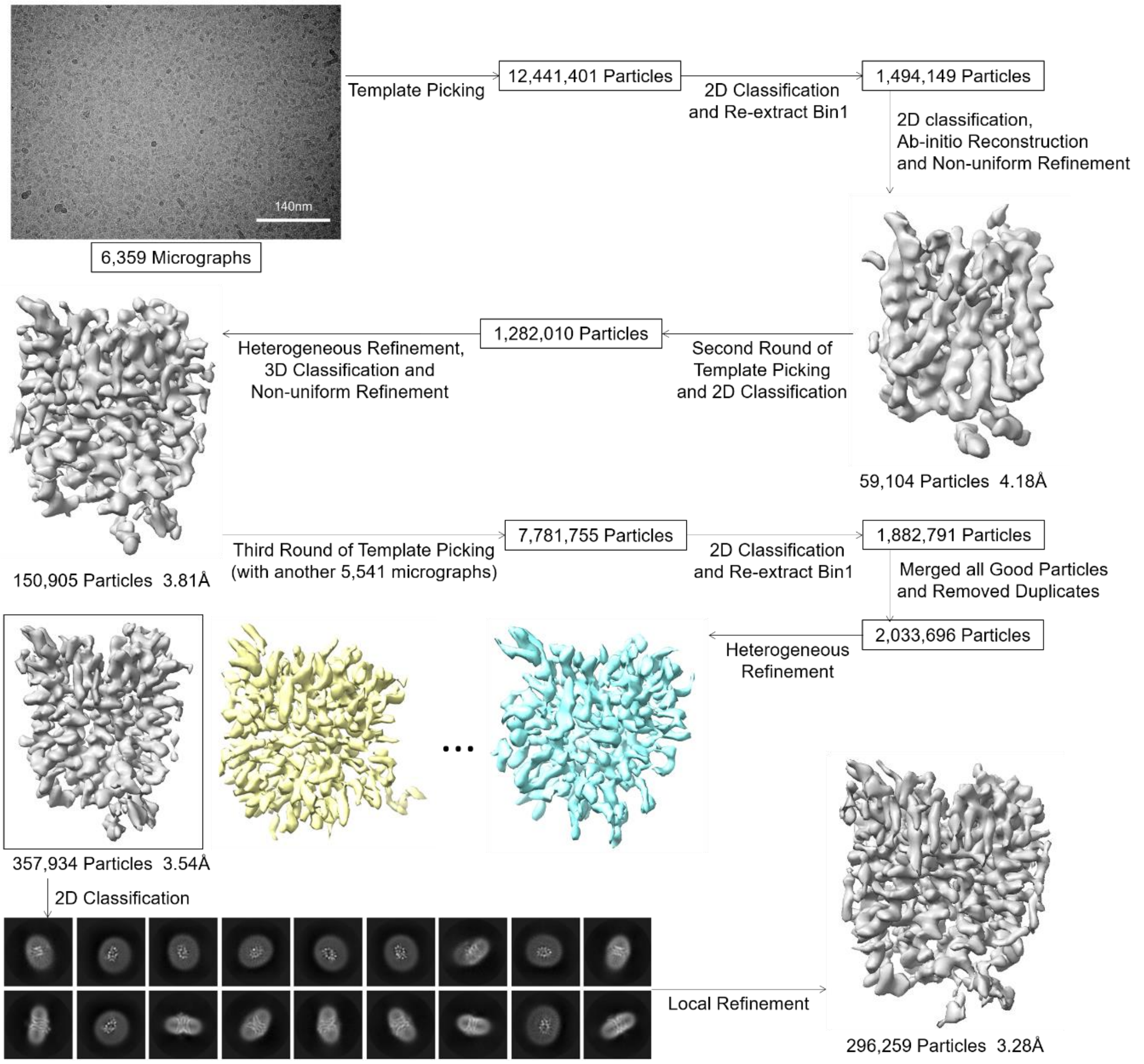

Supplementary Fig. 3 | Flowchart for EM data processing of GLUT9-API. Details can be found in Materials and Methods. A similar data processing protocol was used for GLUT9-UA.

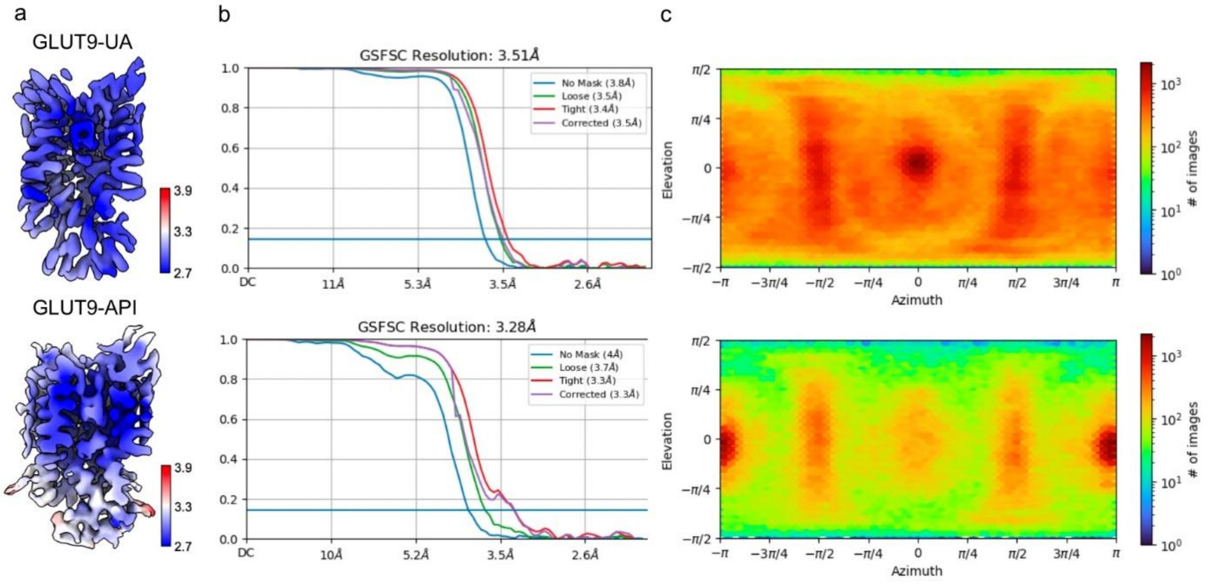

Supplementary Fig. 4 | Cryo-EM analysis of GLUT9-API and GLUT9-UA. a, Local resolution distribution of the cryo-EM maps for GLUT-UA and GLUT9 -API. Local resolutions were estimated with cryoSPARC [2] and represented in ChimeraX [3]. **b,** Gold standard Fourier shell correlation (FSC) curves for the 3D reconstruction calculated in cryoSPARC. **c,** Angular distributions of the particles in the final refinement.

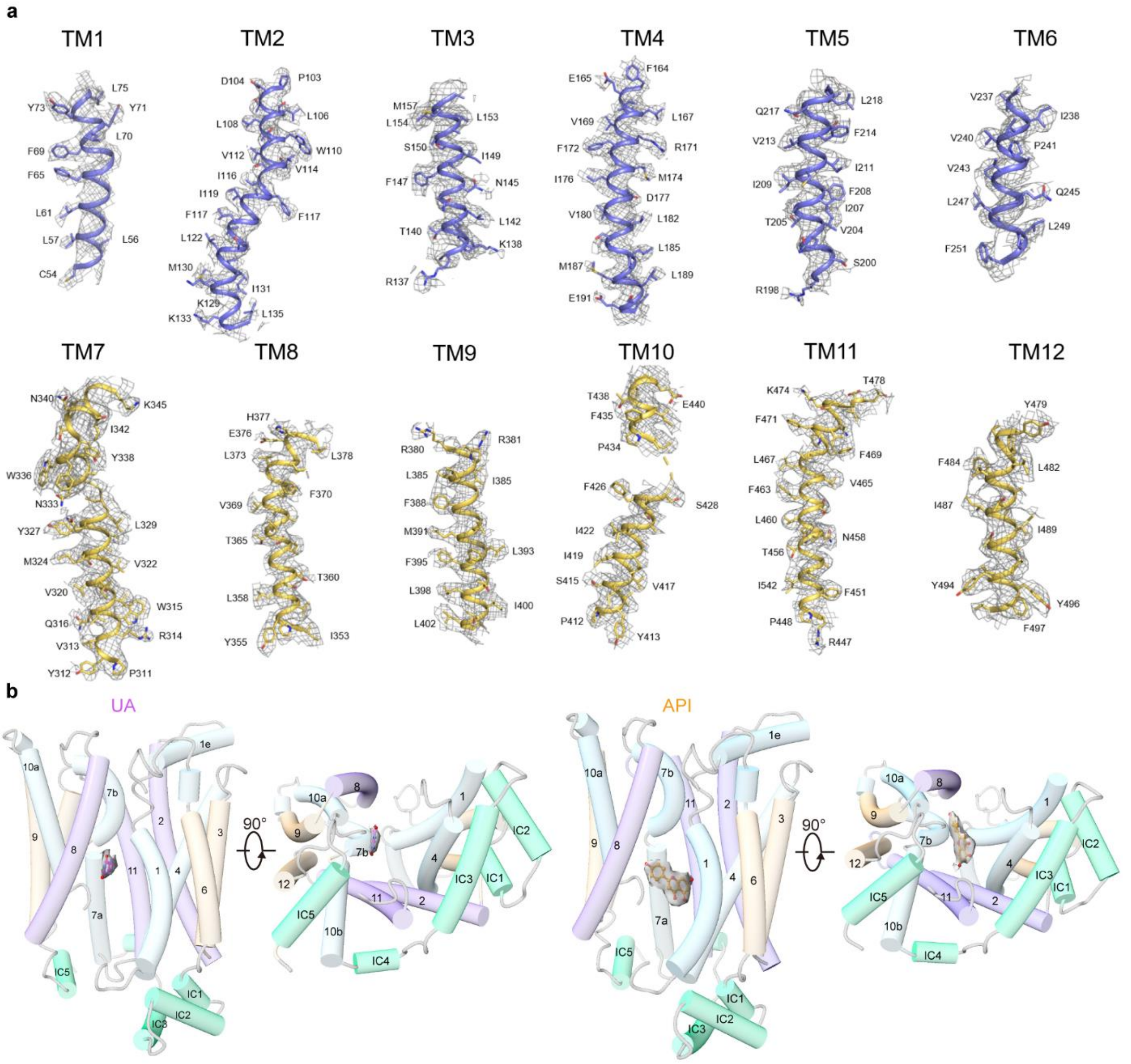

Supplementary Fig. 5 | EM maps of the transmembrane segments and ligands in the GLUT9 complexes. **a,** The densities for the transmembrane segments are isolated from GLUT9-API complex. The bulky residues used to validate the sequence assignment are labeled. The maps were prepared in PyMol [4] and contoured at 4-5 σ. **b**, The corresponding densities for UA and API. For virtual clarity, they are shown in the same views and TM5 is hidden. The maps were prepared in ChimeraX.

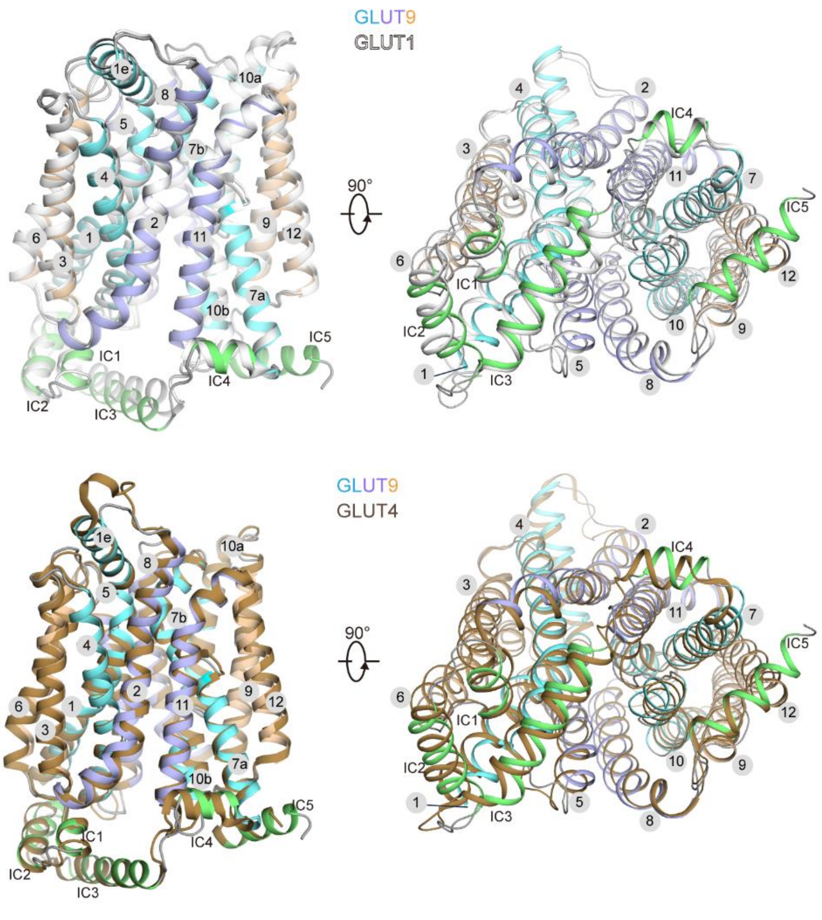

Supplementary Fig. 6 | The overall conformation of GLUT9 is similar to that of GLUT1 and GLUT4. The structure of GLUT9 can be superimposed with GLUT1 [5] (PDB code: 4PYP) and GLUT4 [6] (PDB code: 7WSM) with root-mean-square deviation (RMSD) values of 1.24 Å among 396 Cα atoms and 1.19 Å among 413 Cα atoms, respectively.

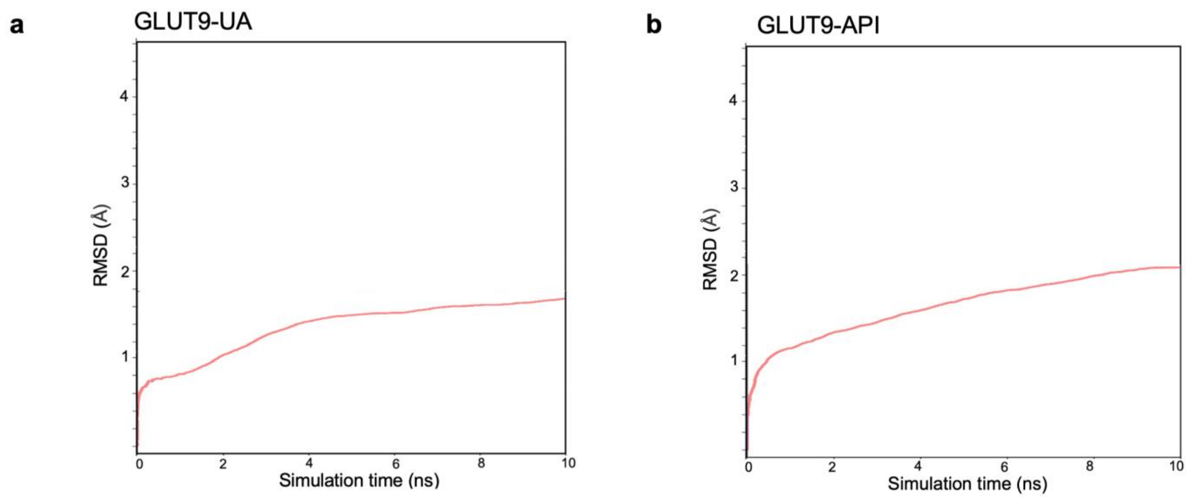

Supplementary Fig. 7 | Binding pose validation with the metadynamics simulations. a, Average RMSD of urate in GLUT9-UA during the 10 × 10 ns metadynamics runs. **b,** Average RMSD of glucose in GLUT9-API during the 10 × 10 ns metadynamics runs. The PoseScore provided by BPMD for GLUT9-UA and GLUT9-API are 1.78 and 2.16 Å, respectively. This indicates that the ligands have been accurately modeled and are well-refined in the cryo-EM structures

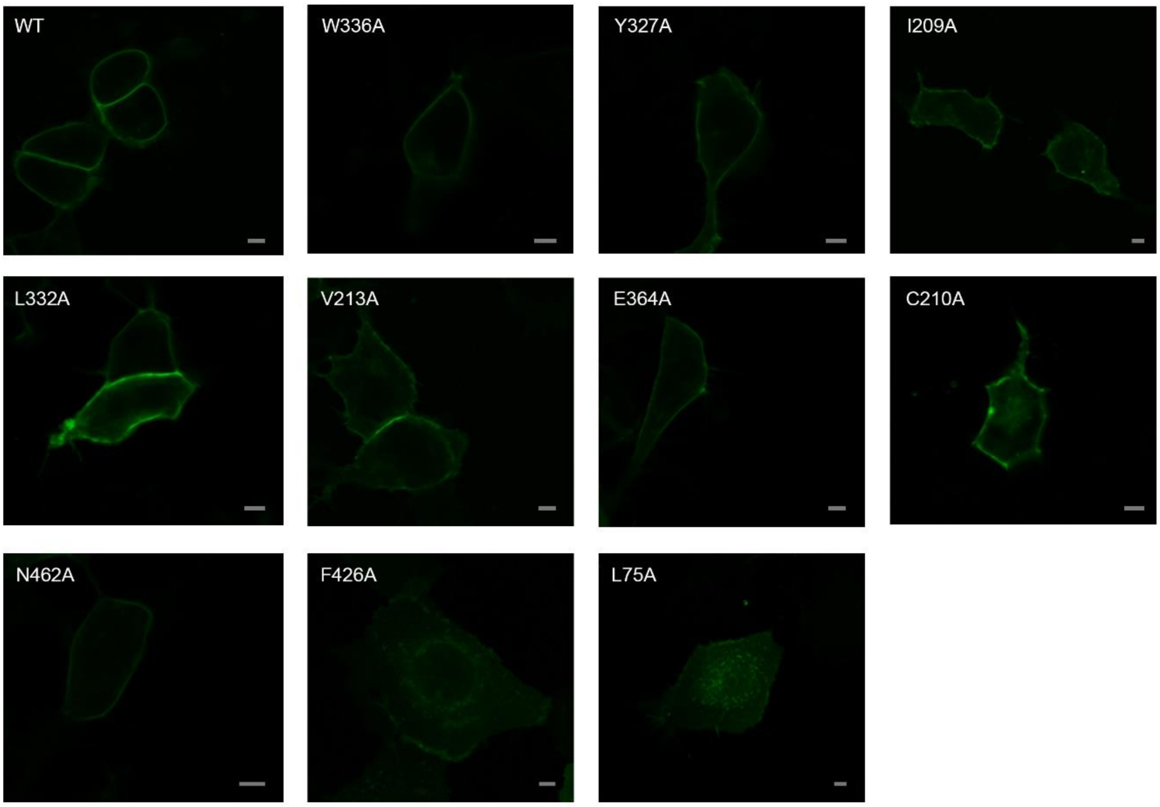

Supplementary Fig. 8 | GLUT9 variations expression in HEK293T cells. Confocal imaging of GFP-tagged GLUT9 variants demonstrated that, with the exception of L75A, all mutations had no effect on protein expression and trafficking. L75A predominantly localized to intracellular membranes and was not detectable via electrophysiology studies. The varying lengths of the gray bars corresponded to 5 μm in all the indicated variant images.

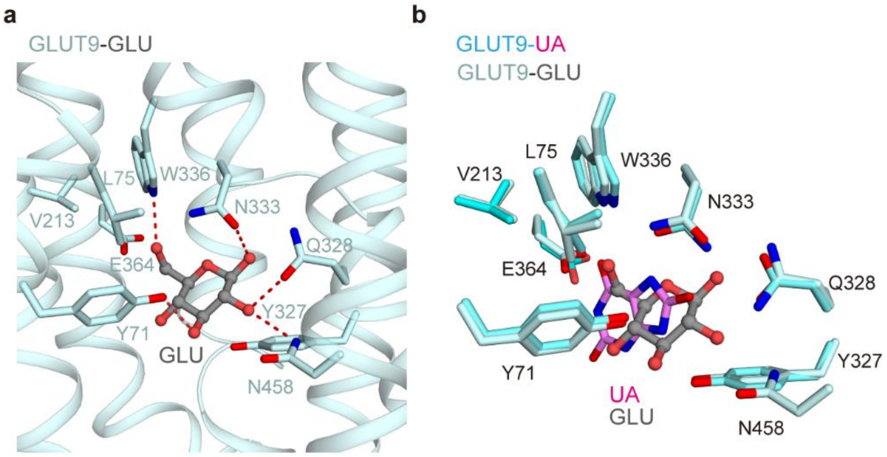

Supplementary Fig. 9 | GLUT9 or glucose should undergo conformational change or rotation to guarantee their binding. **a,** GLUT9-GLU binding mode by molecular docking. L75, Y71, V213, Y327, Q328, N333, W336 and E364, which are involved in glucose binding of GLUT9 are shown in sticks. The direct H-bonds are indicated by red dashed lines. **b,** Superimposition of the central binding pockets of GLUT9-UA structure and GLUT9-GLU docking model. The two structures are shown in a view identical to Fig. 2b.

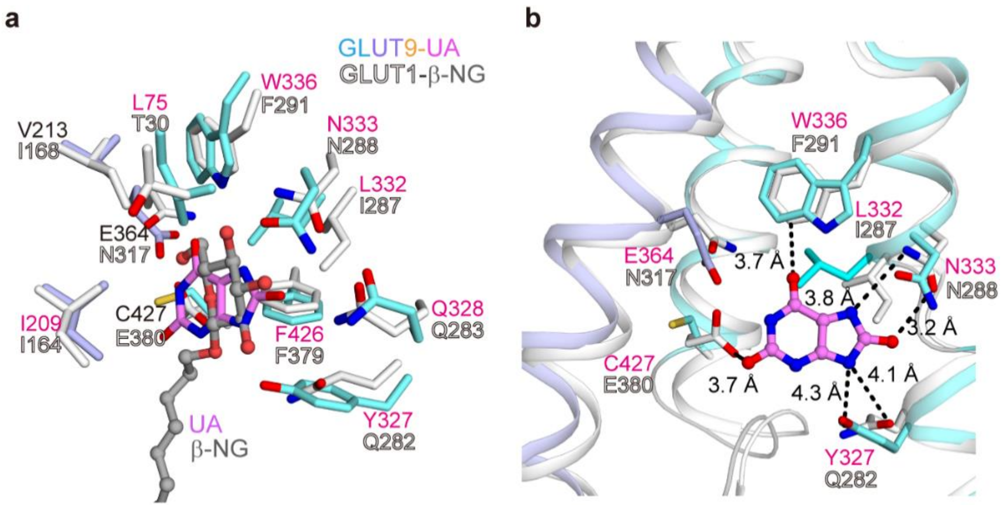

Supplementary Fig. 10 | Molecular basis for urate cannot be recognized by GLUT1. a, Superimposition of the central binding pockets of GLUT9 and GLUT1. The two structures are shown in a view identical to Fig. 2b. The residues involved in interactions with ligands are shown in sticks. **b,** GLUT1 lacks the majority of the hydrogen bonds with urate that are present in GLUT9. Different counterpart residues of GLUT9 and GLUT1, and N333/N288 are shown in sticks. The distances between urate and the indicated residues in GLUT1 are represented by black dashes.

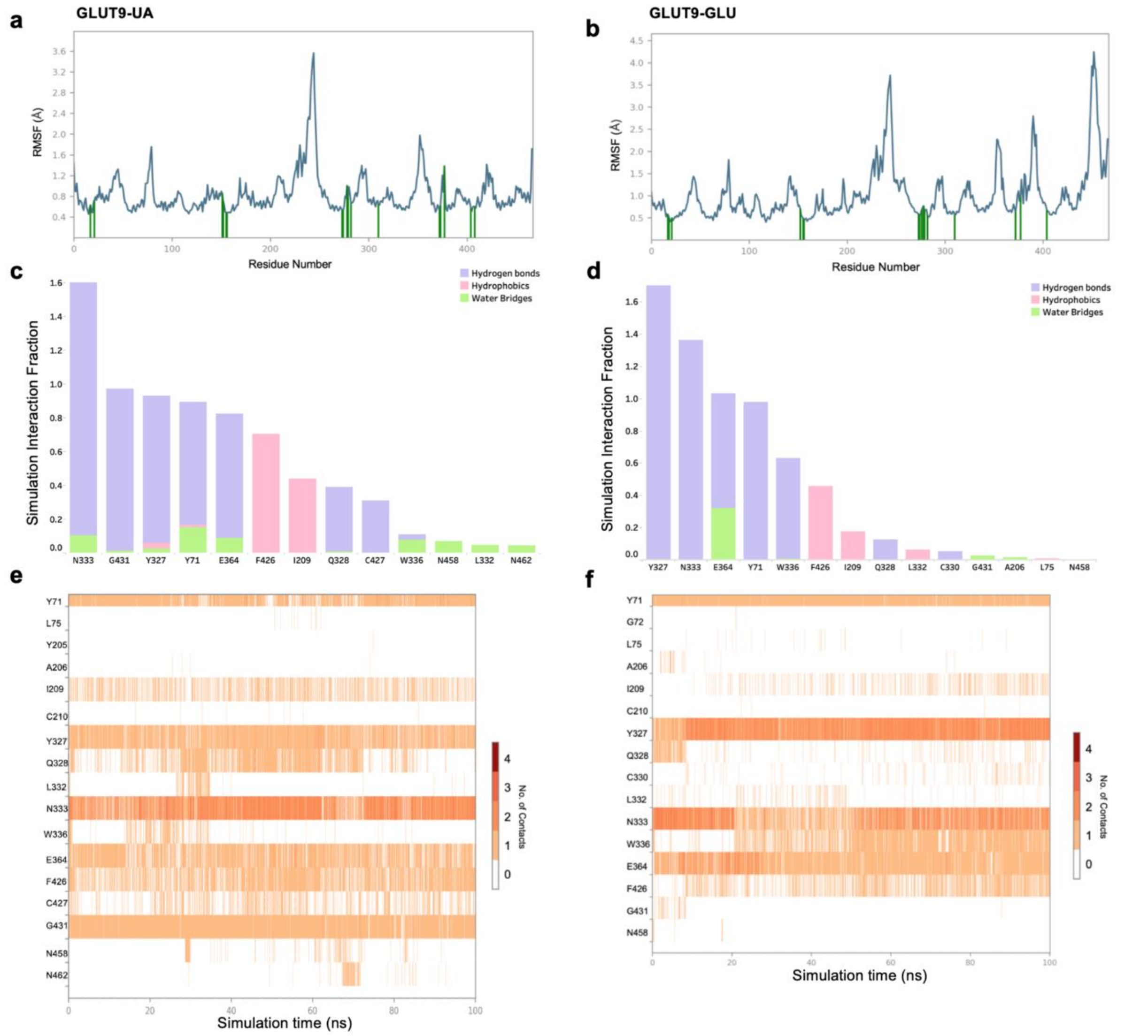

Supplementary Fig. 11 | Comparison of the interactions of GLUT9-UA and GLUT9-GLU. **a/b**, Root mean square fluctuation (RMSF) computed for the GLUT9-UA (a) and GLUT-GLU (b) complexes throughout the simulation. The residues that interact with ligands are indicated in green. **c/d,** GLUT9 interactions with urate (c) and glucose (d) monitored throughout the simulation trajectories. The interactions are clustered by type and shown in the bar diagram, including H-bonds (purple), hydrophobic (pink), and water bridges (green). Since the H-bond can function as both a proton donor and acceptor during the simulation, its proportion may exceed 1. **e/f,** The number of contacts formed between GLUT9 with urate (e) and glucose (f) during 100 ns.

Supplementary Table 1 | Summary of mutations derived from Type 2 renal hypouricemia (RHU2) in GLUT9.

Variants	Disease description	Function validation	References
L75R	For homozygous individuals: SUA=0.17 ±0.20 mg/dl, FE>150%; For heterozygous individuals: SUA=2.88 ±0.87 mg/dl, FE=13.00 ±6.74%[7]	Reduced activity[7] and protein expression[8]	7,8
T125M	SUA=0.2 mg/dl, FE>150%[9]	Reduced activity[9] and protein expression[8]	8,9
R171C	For homozygous individuals: SUA=0.1-0.2 mg/dl, FE=88.8-157%; For heterozygous individuals: SUA=3.8-4.9 mg/dl, FE=3.2-6.5%[9]	Reduced activity[9] and protein expression[8]	8,9
R198C	For heterozygous individuals :SUA=2.1 mg/dl[10]	Reduced activity[8,10]	8,10
G216R	For homozygous individuals: SUA=0.5 mg/dl, FE=45.8%[11]	No protein expressed[8]	8,11
N333S	For heterozygous individuals combined with G216R: SUA=0.67 mg/dl, FE=93%[11]	Reduced activity[8]	8,11
R380W	For heterozygous individuals: SUA=1.5-2.7 mg/dl, FE=14.6-15.7% [10]	Reduced activity[10]	10

SUA: Serum urate concentration; FE: fractional excretion. The normal range for SUA are 3.5-7.2 mg/dl and 2.6-6.0 mg/dl in males and females respectively, for FE is 5-10%. All the mutations in the table are homozygous if not indicated.

Supplementary Table 2 | Summary of inhibitors of GLUT9.

Inhibitors	Target proteins	Sources	IC_{50} of GLUT9	References
Benzbromarone	GLUT9, URAT1	Clinical drugs	28.58 ± 0.071 μM	12
Probenecid	GLUT9, URAT1		731.40 ± 0.047 μM	12
Apigenin	GLUT9, URAT1	Natural products	2.63 ± 0.69 μM	13
Resveratrol	GLUT9		68.77 ± 11.18 μM	14
Isobavachin	GLUT9, URAT1, OAT1, OAT3, ABCG2, XOD		1.12 ± 0.26 μM	15
Morin	GLUT9, URAT1, OAT1, OAT3, ABCG2, XOD		18.44 ± 3.14 μM	15
Baicalein	GLUT9, URAT1, XOD		30.17 ± 8.68 μM	16
KPH2f	GLUT9, URAT1	Synthesized	9.37 ± 7.10 μM	17
CDER167	GLUT9, URAT1		91.55 ± 15.28 μM	18
Tranilast	GLUT9, URAT1, OAT4, OAT10	Other drugs	~ 15.6 μM	19

Supplementary Table 3 | Statistics for data collection and model refinement.

	GLUT9-UA	GLUT9-API
**Data collection and processing**		
Magnification	64,000	81,000
Voltage (kV)	300	300
Electron dose (e-/Å^2)	50	50
Defocus range (μm)	-1.8~-1.5	-1.8~-1.5
Pixel size (Å)	1.0979	1.0825
Symmetry	C1	C1
Initial particle images	7,785,838	18,043,281
Final particle images	810,830	296,259
Map resolution (Å)	3.51	3.28
FSC threshold	0.143	0.143
**Refinement**		
Initial model used	AlphaFold prediction: AF-Q9NRM0-F1-model_v4	
Map sharpening *B* factor (Å^2)	214.1	178.6
Model composition		
Non-hydrogen atoms	3565	3606
Protein residues	467	467
Ligands	1	1
B factors (Å^2)		
Protein	46.17	117.62
Ligand	45.27	110.47
R.m.s deviations		
Bond lengths (Å)	0.002	0.004
Bond angles (°)	0.602	0.661
Validation		
MolProbity score	1.72	1.73
Clashscore	8.88	10.23
Poor rotamers (%)	0.00	0.00
Ramachandran plot		
Favored (%)	96.34	96.77
Allowed (%)	3.66	3.23
Disallowed (%)	0	0

Supplemental References:

1 Larkin, M. A. *et al.* Clustal W and Clustal X version 2.0. *Bioinformatics* **23**, 2947-2948, doi:10.1093/bioinformatics/btm404 (2007).

2 Punjani, A., Rubinstein, J. L., Fleet, D. J. & Brubaker, M. A. cryoSPARC: algorithms for rapid unsupervised cryo-EM structure determination. *Nat Methods* **14**, 290-296, doi:10.1038/nmeth.4169 (2017).

3 Pettersen, E. F. *et al.* UCSF Chimera--a visualization system for exploratory research and analysis. *J Comput Chem* **25**, 1605-1612, doi:10.1002/jcc.20084 (2004).

4 DeLano, W. L. The PyMOL Molecular Graphics System. www.pymol.org (2002).

5 Deng, D. *et al.* Crystal structure of the human glucose transporter GLUT1. *Nature* **510**, 121-125, doi:10.1038/nature13306 (2014).

6 Yuan, Y. *et al.* Cryo-EM structure of human glucose transporter GLUT4. *Nat Commun* **13**, 2671, doi:10.1038/s41467-022-30235-5 (2022).

7 Dinour, D. *et al.* Homozygous SLC2A9 mutations cause severe renal hypouricemia. *J Am Soc Nephrol* **21**, 64-72, doi:10.1681/ASN.2009040406 (2010).

8 Ruiz, A., Gautschi, I., Schild, L. & Bonny, O. Human Mutations in SLC2A9 (Glut9) Affect Transport Capacity for Urate. *Front Physiol* **9**, 476, doi:10.3389/fphys.2018.00476 (2018).

9 Dinour, D. *et al.* Two novel homozygous SLC2A9 mutations cause renal hypouricemia type 2. *Nephrol Dial Transplant* **27**, 1035-1041, doi:10.1093/ndt/gfr419 (2012).

10 Matsuo, H. *et al.* Mutations in glucose transporter 9 gene SLC2A9 cause renal hypouricemia. *Am J Hum Genet* **83**, 744-751, doi:10.1016/j.ajhg.2008.11.001 (2008).

11 Stiburkova, B., Taylor, J., Marinaki, A. M. & Sebesta, I. Acute kidney injury in two children caused by renal hypouricaemia type 2. *Pediatr Nephrol* **27**, 1411-1415, doi:10.1007/s00467-012-2174-0 (2012).

12 Chen, Y. *et al.* Characterizations of the Urate Transporter, GLUT9, and Its Potent Inhibitors by Patch-Clamp Technique. *SLAS Discov* **26**, 450-459, doi:10.1177/2472555220949501 (2021).

13 Li, Y. *et al.* Apigenin ameliorates hyperuricemic nephropathy by inhibiting URAT1 and GLUT9 and relieving renal fibrosis via the Wnt/beta-catenin pathway. *Phytomedicine* **87**, 153585, doi:10.1016/j.phymed.2021.153585 (2021).

14 Li, L. *et al.* Resveratrol, a novel inhibitor of GLUT9, ameliorates liver and kidney injuries in a D-galactose-induced ageing mouse model via the regulation of uric acid metabolism. *Food Funct* **12**, 8274-8287, doi:10.1039/d1fo00538c (2021).

15 Zhao, Z. *et al.* Pharmacological evaluation of a novel skeleton compound isobavachin (4?,7-dihydroxy-8-prenylflavanone) as a hypouricemic agent: Dual actions of URAT1/GLUT9 and xanthine oxidase inhibitory activity. *Bioorg Chem* **133**, doi:ARTN 106405 10.1016/j.bioorg.2023.106405 (2023).

16 Chen, Y. *et al.* Baicalein alleviates hyperuricemia by promoting uric acid excretion and inhibiting xanthine oxidase. *Phytomedicine* **80**, 153374, doi:10.1016/j.phymed.2020.153374 (2021).

17 Zhao, Z. *et al.* Discovery of novel verinurad analogs as dual inhibitors of URAT1 and GLUT9 with improved Druggability for the treatment of hyperuricemia. *European Journal of Medicinal Chemistry* **229**, doi:ARTN 114092 10.1016/j.ejmech.2021.114092 (2022).

18 Zhao, Z. A. *et al.* CDER167, a dual inhibitor of URAT1 and GLUT9, is a novel and potent uricosuric candidate for the treatment of hyperuricemia. *Acta Pharmacol Sin* **43**, 121-132, doi:10.1038/s41401-021-00640-5 (2022).

19 Mandal, A. K., Mercado, A., Foster, A., Zandi-Nejad, K. & Mount, D. B. Uricosuric targets of tranilast. *Pharmacol Res Perspect* **5**, e00291, doi:10.1002/prp2.291 (2017).
