# Supplementary material for: Structural basis for urate recognition and apigenin inhibition of human GLUT9
Source: Nat Commun. 2024 Jun 12;15:5039. doi: 10.1038/s41467-024-49420-9 (PMC11169512; doi:10.1038/s41467-024-49420-9)
Supplement: Supplementary file 2 — Peer Review File [file 41467_2024_49420_MOESM2_ESM.pdf]

nature portfolio

Peer Review File

Structural basis for urate recognition and apigenin inhibition of human GLUT9

Note: Figure 1 in the author rebuttal is derived from Benn CL, Dua P, Gurrell R, Loudon P, Pike A, Storer RI and Vangjeli C (2018) Physiology of Hyperuricemia and Urate-Lowering Treatments. Front. Med. 5:160. doi: 10.3389/fmed.2018.00160. Distributed under the terms of the Creative Commons Attribution License (CC BY): https://creativecommons.org/licenses/by/4.0/

Open Access This file is licensed under a Creative Commons Attribution 4.0 International License, which permits use, sharing, adaptation, distribution and reproduction in any medium or format, as long as you give appropriate credit to the original author(s) and the source, provide a link to the Creative Commons license, and indicate if changes were made. In the cases where the authors are anonymous, such as is the case for the reports of anonymous peer reviewers, author attribution should be to 'Anonymous Referee' followed by a clear attribution to the source work. The images or other third party material in this file are included in the article's Creative Commons license, unless indicated otherwise in a credit line to the material. If material is not included in the article's Creative Commons license and your intended use is not permitted by statutory regulation or exceeds the permitted use, you will need to obtain permission directly from the copyright holder. To view a copy of this license, visit http://creativecommons.org/licenses/by/4.0/.

REVIEWER COMMENTS

Reviewer #1 (Remarks to the Author):

In the manuscript, the authors presented the cryo-electron microscopy structures of human GLUT9 bound with urate and its inhibitor Apigenin, and revealed the structural basis for substrate preference of urate over glucose in GLUT9. This is a very good job. It would be of great significance for the development of GLUT9-targeting uricosuric drugs. However, there are some important issues to be revised:
1.Uric acid is an weak acid (pKa=5.5). Does GLUT9 transport uric acid in its ionized or non-ionized form (or both) under physiological conditions? I noticed that the GLUT9 structures were prepared at pH 6.0 instead of pH7.4.What is the status of uric acid in this study?
2.In the part of whole-cell patch-clamp recording of GLUT9, why the bath solution was adjusted to pH5.5?
3.The author has identified multiple binding sites of GLUT9 with UA/API, but these sites appear slightly different between API and UA. Which sites do you think are more critical for uric acid and/or API binding? I suggest highlighting and discussing residues that contribute more to substrate/inhibitor binding, as this information would be helpful for the development of GLUT9-targeting uricosuric drugs.
4.GLUT9 can transport glucose or fructose in addition to uric acid, so it is important to understand the specific binding sites of GLUT9 for uric acid compared to glucose/fucose. I noticed that the author has compared the structure of GLUT1/4 with GLUT9. Undoubtedly, it is pivotal in elucidating the distinctions between GLUT9 and GLUT1/4. Would it be possible to prepare crystal structure of GLUT9 in complex with glucose? Also, docking or MD simulation of GLUT9 with glucose were also convincing and helpful.
5.Why choose API? Has the author tried other commercially available GLUT9 inhibitors that have been reported, such as benzbromarone and tranliast? I think it would be worthwhile to analysis a few more structures and compare their differences.
6. In supplementary Figure 7, the authors utilized electrophysiology to confirm the urate binding residues, and the findings demonstrate the loss of function in all variants. However, I would like to know whether these mutations affected their membrane protein expression and membrane trafficking?
7. Consistent abbreviations for amino acids between figures and texts throughout the manuscript, such as replacing Thr125Met with T125M and Leu75 with L75, are preferable.
8. I suggest the authors emphasize the study's significance in relation to GLUT9 inhibitor design. With the clarification of API's mechanism of action, it is necessary to provide strategies for structural modifications.

Reviewer #2 (Remarks to the Author):

In this manuscript, Pan and co-workers described the structural and functional characterization of GLUT9, a human high-capacity urate transporter, in order to understand its transport and inhibition mechanisms. Using cryo-EM, the authors directly determined extramembrane domain-less GLUT9 in an inward-facing conformation, with substrate urate or inhibitor apigenin at 3.3~3.5 Å resolution. Apigenin partially overlaps with urate in the central binding cavity, suggesting a competitive inhibitory effect. The structural observations were also complemented with whole-cell patch-clamp recording assays upon mutagenesis of critical residues lining the binding pocket. The authors deduced the molecular basis of GLUT9's substrate preference for urate over glucose, by comparing it to the homologous glucose transporter GLUT1.
Given the potential of GLUT9 as a promising target for gout treatment, current research provides important clue for the development of specific inhibitors. In my opinion, the manuscript can be improved by addressing the following comments:

- The precise modelling of ligands is at the heart of interaction network analysis. Given the moderate 3.5 Å map resolution and pseudo-symmetric molecular shape of urate, it is recommended to validate

the pose using other means, such as molecular dynamics simulation analysis.
- Transport activity analysis on mutating the pocket residues is informative, however, it is not necessarily tightly correlated with ligand interactions. It is worthwhile to measure the affinity variations directly.
- Biochemical characterization, such as evaluating urate binding affinity or transport activity when changing GLUT9 pocket residues to GLUT1 counterparts, can deepen the discussion of ligand preference, which is now based entirely on structural comparisons between GLUT9 and GLUT1.

Summary of revisions:

We are grateful to the positive comments from both reviewers, acknowledging the significance of our study. Their constructive suggestions have been immensely valuable in improving the manuscript. Before addressing each reviewer's comments in detail, we would like to provide a summary of the major changes we have made.

1. In light of the critical input from both reviewers, we have conducted molecular dynamics (MD) simulations and molecular docking to investigate the ligand preference of both GLUT9 and the accuracy of both the structures. The results of these simulations have been included in Figure 2d, and supplementary Figures 7, 9 and 11.

2. In response to the suggestions from reviewer #2, we have performed binding assays (MST experiments) specifically examining the interactions of urate and API with GLUT9, and the GLUT9 substrate preference. The corresponding results have been incorporated into Figure 2f, Figure 3e and Figure 4e.

3. To address the concerns raised by reviewer #1 regarding the protein expression and trafficking of the different variations of GLUT9, we have conducted confocal imaging experiments. The results of these experiments are now presented in supplementary Figure 8.

4. Following the comment from reviewer #1, we have added a supplementary Table 1, which summarizes the inhibitors of GLUT9 reported in the literatures. Additionally, we have included further discussions in the relevant section pertaining to drug optimization.

These major changes and additions have strengthened the manuscript in accordance with the reviewers' suggestions, improving the overall quality and comprehensiveness of our study.

Reviewer #1:

Reviewer #1 fully recognized the significance of our study. They raised some constructive and specific suggestions that are addressed below.

1.Uric acid is a weak acid (pKa=5.5). Does GLUT9 transport uric acid in its ionized or non-ionized form (or both) under physiological conditions? I noticed that the GLUT9 structures were prepared at pH 6.0 instead of pH7.4. What is the status of uric acid in this study?

That's an excellent question! The existence of ionized form of uric acid at a solution pH of 5.5 allows us to detect the ion current in our electrophysiological study. Based on the findings depicted in Figure 1 of Benn, *et al.*, *Front Med (Lausanne)*. 2018, it is evident that approximately 98-99% of uric acid becomes ionized under physiological conditions at a pH of 7.4. However, due to the limited resolution of the structural analysis, the hydrogen atom is not clearly discernible. Consequently, we can infer that GLUT9 is capable of transporting the ionized form of uric acid, but its ability to transport the non-ionized form remains unknown.

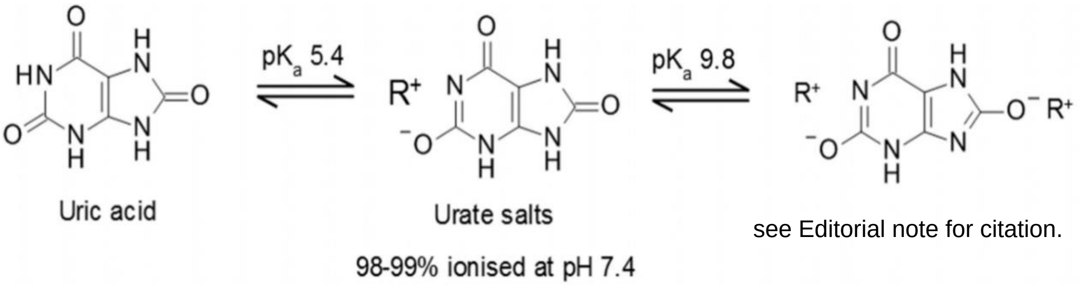

In our study, the pH was 6.0, which is suitable for protein stabilization and it is higher than 5.5, our assumption is that the substrate is partially ionized in our study.

Thank you for raising this point. It has prompted us to delve deeper into the transport mechanism and consider additional aspects related to the ionization of the substrate.

2. In the part of whole-cell patch-clamp recording of GLUT9, why the bath solution was adjusted to pH5.5?

Anzai *et al.*, *JBC*, 2008 and Chen *et al.*, *SLAS Discovery*, 2021 reported that pH5.5 is better than pH7.4 when conduct the transport activity. The uptake amount at pH 5.5 was approximately 1.5 times higher than that at pH7.4.

Specifically, Chen et al. established a well-characterized electrophysiology system utilizing HEK293 cells overexpressing GLUT9. In their study, they set

electrophysiological experiments to measure the conductance of urate at various pH levels. Interestingly, their results indicated that pH5.5 yielded the largest peak current. And this is consistent with the results in Anzai *et al.*, *JBC*, 2008. That is the reason that we choose pH5.5 directly.

3.The author has identified multiple binding sites of GLUT9 with UA/API, but these sites appear slightly different between API and UA. Which sites do you think are more critical for uric acid and/or API binding? I suggest highlighting and discussing residues that contribute more to substrate/inhibitor binding, as this information would be helpful for the development of GLUT9-targeting uricosuric drugs.

Thank you for raising this point. It is true that the UA and API binding sites in GLUT9 exhibit slight differences. However, it should be noted that GLUT9 can bind to either of them as these binding sites overlap. API functions as a competitive inhibitor. Through MST analysis, we investigated the binding residues and found that some of them play crucial roles in these interactions. The findings and discussions regarding these interactions have been included in the relevant session.

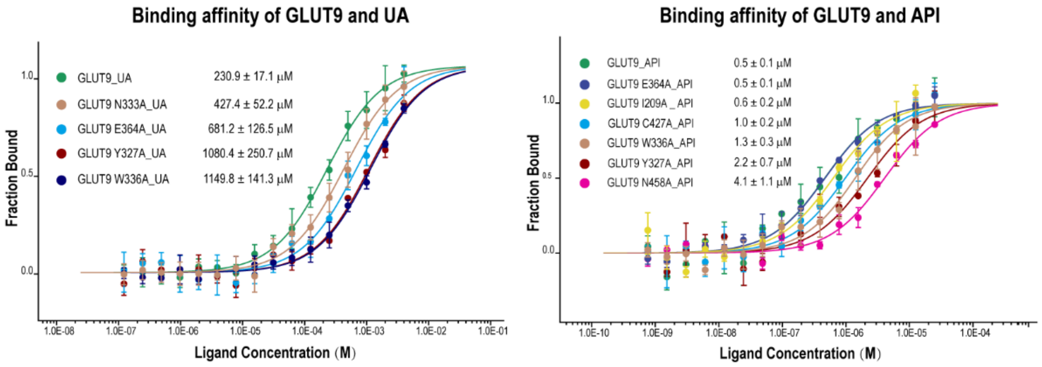

4.GLUT9 can transport glucose or fructose in addition to uric acid, so it is important to understand the specific binding sites of GLUT9 for uric acid compared to glucose/fucose. I noticed that the author has compared the structure of GLUT1/4 with GLUT9. Undoubtedly, it is pivotal in elucidating the distinctions between GLUT9 and GLUT1/4. Would it be possible to prepare crystal structure of GLUT9 in complex with glucose? Also, docking or MD simulation of GLUT9 with glucose were also convincing and helpful.

We appreciate your constructive suggestion. Solving the complex structure of GLUT9 and glucose with binding affinity in the millimolar range can be very challenging. To address this limitation, we reached out to our collaborator who specializes in molecular dynamics (MD) simulations. They conducted MD simulations and molecular docking specifically focusing on the binding of GLUT9 with glucose or urate. The results of these simulations are now presented in Supplementary Figures 9 and 11. This addition provides valuable

insights into the interaction between GLUT9 and glucose, which also shows that GLUT9 or the substrate glucose should undergo slight conformational changes to guarantee the binding.

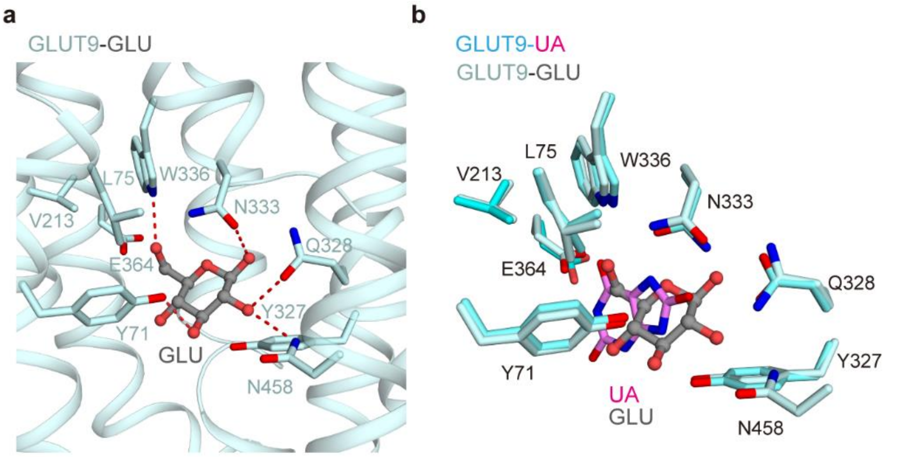

Supplementary Fig. 9

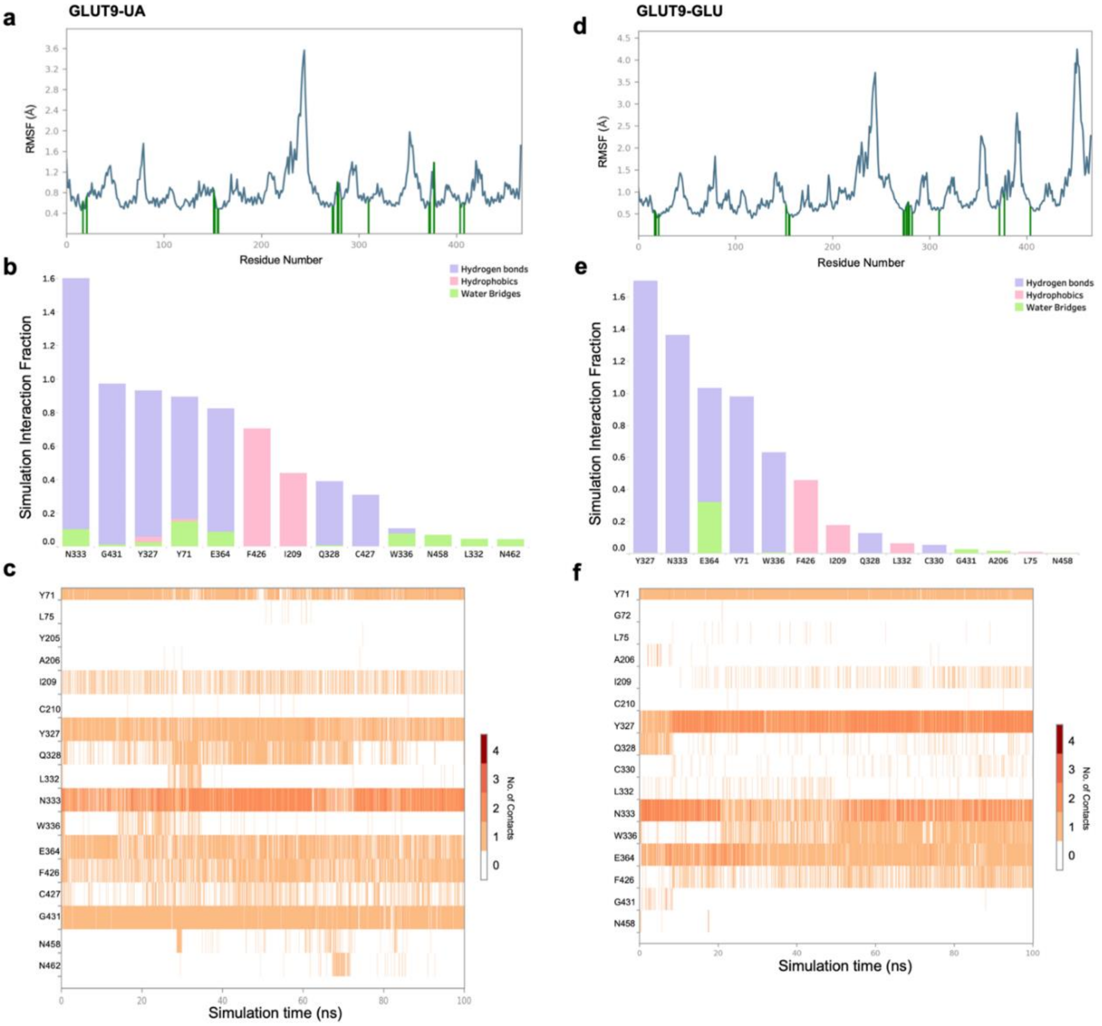

Supplementary Fig. 11

5.Why choose API? Has the author tried other commercially available GLUT9 inhibitors that have been reported, such as benzbromarone and tranliast? I think it would be worthwhile to analysis a few more structures and compare their differences.

We have taken this comment into consideration and have included a comprehensive summary of inhibitors that bind with GLUT9 from various papers. This information can now be found in supplementary Table 1. Among these inhibitors, apigenin stands out with fewer binding targets and higher binding affinity compared to others. It is worth noting that we also attempted to determine the complex structures of GLUT9 with benzbromarone or resveratrol. However, the density for benzbromarone was weak, and there was almost no density for resveratrol after data processing.

Inhibitors	Target proteins	Sources	IC_{50} of GLUT9	References
Benzbromarone	GLUT9, URAT1	Clinical drugs	28.58 ± 0.071 μM	7
Probenecid	GLUT9, URAT1		731.40 ± 0.047 μM	7
Apigenin	GLUT9, URAT1	Natural products	2.63 ± 0.69 μM	8
Resveratrol	GLUT9		68.77 ± 11.18 μM	9
Isobavachin	GLUT9, URAT1, OAT1, OAT3, ABCG2, XOD		1.12 ± 0.26 μM	10
Morin	GLUT9, URAT1, OAT1, OAT3, ABCG2, XOD		18.44 ± 3.14 μM	10
Baicalein	GLUT9, URAT1, XOD		30.17 ± 8.68 μM	11
KPH2f	GLUT9, URAT1	Synthesized	9.37 ± 7.10 μM	12
CDER167	GLUT9, URAT1		91.55 ± 15.28 μM	13
Tranilast	GLUT9, URAT1, OAT4, OAT10	Other drugs	~ 15.6 μM	14

Supplementary Table 1

6. In supplementary Figure 7, the authors utilized electrophysiology to confirm the urate binding residues, and the findings demonstrate the loss of function in all variants. However, I would like to know whether these mutations affected their membrane protein expression and membrane trafficking?

We appreciate your valuable suggestion. To address concerns regarding protein expression and trafficking of GLUT9 variants, we have made significant efforts. Firstly, we generated a GFP fusion protein at the N terminus of GLUT9 to facilitate visualization. We then performed electrophysiology experiments to confirm the functionality of this construct. Additionally, we introduced single point mutations to the wildtype GLUT9 and conducted confocal imaging analysis. The results revealed that nearly all of the variations exhibited plasma membrane expression except for L75A which located on the intracellular

membranes. These findings have been included in supplementary Figure 8 in the revised version of the manuscript.

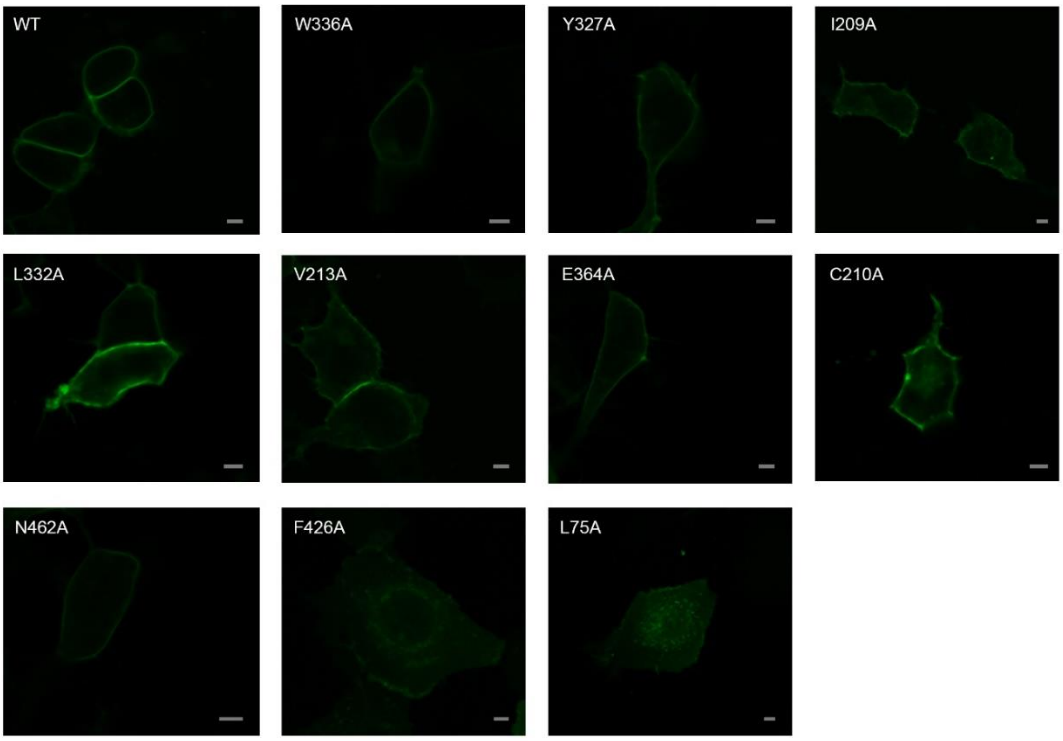

Supplementary Fig. 8

7. Consistent abbreviations for amino acids between figures and texts throughout the manuscript, such as replacing Thr125Met with T125M and Leu75 with L75, are preferable.

We are sorry for this oversight, we have checked all the figures and texts, and now they are consistent.

8. I suggest the authors emphasize the study's significance in relation to GLUT9 inhibitor design. With the clarification of API's mechanism of action, it is necessary to provide strategies for structural modifications.

We appreciate your constructive suggestion. In response, we have taken the opportunity to enhance the discussion in the API inhibitory mechanism section with MST results. We have added additional insights and explanations to better elucidate the underlying mechanisms.

We thank this reviewer for carefully reading our manuscript and for their constructive comments!

Reviewer #2:

Reviewer #2 also recognized the significance of our study. He or she raised some constructive suggestions to improve the manuscript that are addressed below.

1. The precise modelling of ligands is at the heart of interaction network analysis. Given the moderate 3.5 Å map resolution and pseudo-symmetric molecular shape of urate, it is recommended to validate the pose using other means, such as molecular dynamics simulation analysis.

We appreciate this valuable suggestion. The binding pose of the urate and apigenin to GLUT9 from the cryo-EM structures were initially verified using binding pose metadynamics (BPMD) simulations. Since the PoseScore offered by BPMD for the GLUT9-UA and GLUT9-API is 1.78 Å and 2.16 Å, repectively, it signifies that the ligands have been precisely modeled and are well-defined in the Cryo-EM structures (Supplementary Fig. 7a).

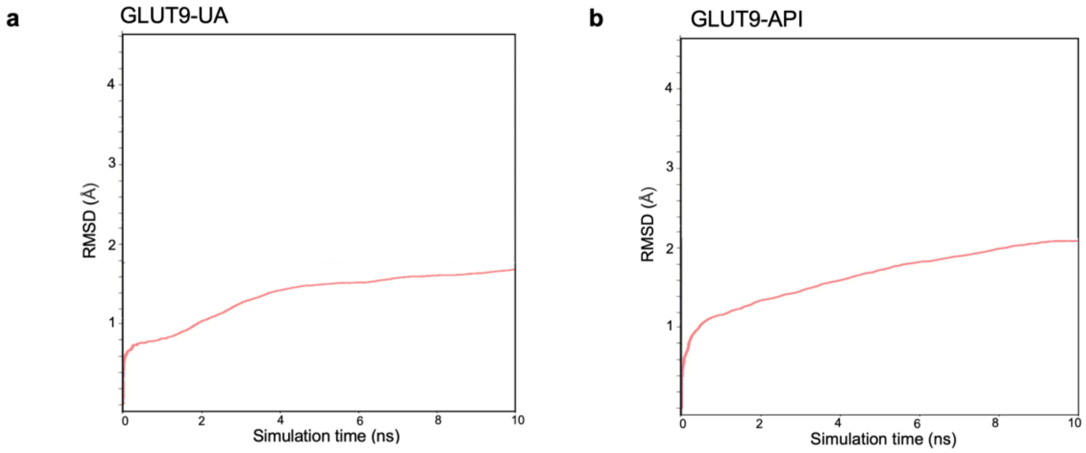

Supplementary Fig. 7

2. Transport activity analysis on mutating the pocket residues is informative, however, it is not necessarily tightly correlated with ligand interactions. It is worthwhile to measure the affinity variations directly.

This is an excellent suggestion. In order to provide comprehensive data on the binding interactions, we have conducted MST experiments to evaluate the binding of urate and API with GLUT9 variants. Ala substitutions decreased the binding affinity with the ligands significantly. The results of these experiments have been incorporated into our manuscript and are now presented in Figures 2f and 4e.

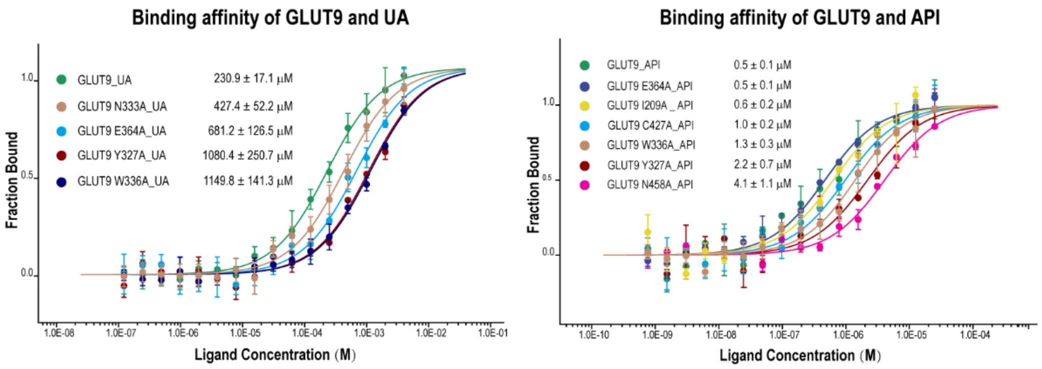

Figures 2f and 4e

3. Biochemical characterization, such as evaluating urate binding affinity or transport activity when changing GLUT9 pocket residues to GLUT1 counterparts, can deepen the discussion of ligand preference, which is now based entirely on structural comparisons between GLUT9 and GLUT1.

Thank you for providing this advice. To validate the ligand preference, we have employed a combination of molecular docking, MD simulations and binding assays using MST. The simulations results have been depicted in Supplementary Figure 9 and 11. For the MST part, the urate binding was detected with the GLUT9 mutants from GLUT1 counterparts. The data is now in Figure 3e.

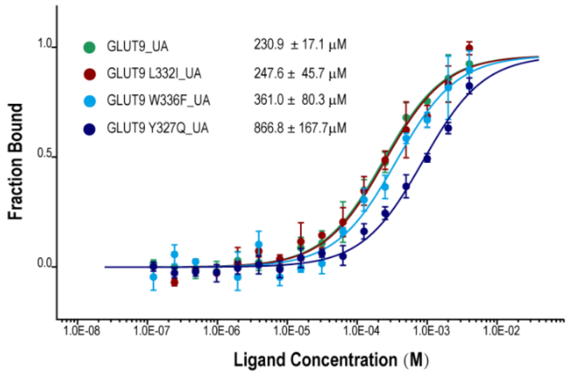

Figure 3e

We thank this reviewer for their constructive suggestions to improve the manuscript quality**.**

REVIEWERS' COMMENTS

Reviewer #1 (Remarks to the Author):

The questions I was concerned about have all been solved or provided with reasonable explanations, and we greatly appreciate the author's revisions to the article. The current version is suitable for publication in Nature Communications.

Reviewer #2 (Remarks to the Author):

Despite the relatively short MD simulation duration (100 ns), the authors have addressed all of my concerns. It is ready for publication at this point.
